# Improved phylogenomic sampling of free-living nematodes enhances resolution of higher-level nematode phylogeny

**DOI:** 10.1186/s12862-019-1444-x

**Published:** 2019-06-13

**Authors:** Ashleigh B. Smythe, Oleksandr Holovachov, Kevin M. Kocot

**Affiliations:** 1 Department of Biology, Virginia Military Institute, 301B Maury-Brooke Hall, Lexington, VA 24450 USA; 2 Department of Zoology, Swedish Museum of Natural History, Box 50007, SE-104 05 Stockholm, Sweden; 3 Department of Biological Sciences and Alabama Museum of Natural History, The University of Alabama, Campus Box 870344, Tuscaloosa, AL 35487 USA

**Keywords:** Nematoda, Phylogenomic, Free-living, Parasitism

## Abstract

**Background:**

Nematodes are among the most diverse and abundant metazoans on Earth, but research on them has been biased toward parasitic taxa and model organisms. Free-living nematodes, particularly from the clades Enoplia and Dorylaimia, have been underrepresented in genome-scale phylogenetic analyses to date, leading to poor resolution of deep relationships within the phylum.

**Results:**

We supplemented publicly available data by sequencing transcriptomes of nine free-living nematodes and two important outgroups and conducted a phylum-wide phylogenomic analysis including a total of 108 nematodes. Analysis of a dataset generated using a conservative orthology inference strategy resulted in a matrix with a high proportion of missing data and moderate to weak support for branching within and placement of Enoplia. A less conservative orthology inference approach recovered more genes and resulted in higher support for the deepest splits within Nematoda, recovering Enoplia as the sister taxon to the rest of Nematoda. Relationships within major clades were similar to those found in previously published studies based on 18S rDNA.

**Conclusions:**

Expanded transcriptome sequencing of free-living nematodes has contributed to better resolution among deep nematode lineages, though the dataset is still strongly biased toward parasites. Inclusion of more free-living nematodes in future phylogenomic analyses will allow a clearer understanding of many interesting aspects of nematode evolution, such as morphological and molecular adaptations to parasitism and whether nematodes originated in a marine or terrestrial environment.

**Electronic supplementary material:**

The online version of this article (10.1186/s12862-019-1444-x) contains supplementary material, which is available to authorized users.

## Background

Nematodes are ubiquitous and diverse metazoans that are found free-living in nearly every terrestrial and aquatic habitat and parasitizing most animals and plants. Fewer than 30,000 species have been described, but the actual diversity of the phylum may be closer to 1 million species [1]. Despite estimates that at least half of all nematodes are free-living [1, 2], most research has focused on parasitic nematodes of medical and agricultural importance. Particularly neglected are the free-living marine nematodes, with only around 6900 species described [3] and no genomes published to date [4]. Of significance, free-living nematodes are generally the most abundant and diverse metazoans of marine sediments [5–8] where they are important as decomposers, predators, food for higher trophic levels [9], and as bioindicators for climate change and ecological disturbance [10–12].

Despite the importance of nematodes as free-living animals and as parasites of humans, livestock, and crops, and despite more than a century of intensive research, certain aspects of their origin and early evolution, such as the branching order near the root of Nematoda, are not yet fully understood [13, 14]. Nematode evolutionary history is particularly interesting because of the diversity of niches they occupy – ranging from the blood and tissues of vertebrate and invertebrate animals, unicellular eukaryotes, all parts of plants, virtually every terrestrial habitat, and all aquatic environments including deep-sea hydrothermal vent communities – is unrivaled in Metazoa [15–18]. Thus, resolving nematode phylogeny, especially the branching order close to the root of the nematode tree, will not only improve our understanding of the origin of economically important groups, but will provide a phylogenetic framework for understanding the underlying key characters (e.g., genomic modifications) corresponding to different nematode lifestyles, advancing all aspects of nematology, from basic evolutionary biology to pathogen control and drug development [19].

Morphology-based hypotheses of higher-level nematode relationships (reviewed by [17, 20–22]) placed emphasis on the presence or absence of a lateral canal excretory system and a number of esophageal features [23, 24]. These characters were interpreted as evidence of two major lineages: a primarily terrestrial (but also including many plant and animal parasites) grouping called Secernentea, and a primarily aquatic grouping called Adenophorea. Subsequent morphological investigations by Andrássy [25] and Malakhov [26] distinguished three main lineages, elevating the primarily aquatic Enoplia and Chromadoria out of Adenophorea and re-classifying most Secernentea as Rhabditia.

The first molecular phylogenetic hypothesis for Nematoda used 18S rDNA [27] and differed substantially from previous morphology-based hypotheses of nematode phylogeny (e.g. [24]). This and subsequent analyses based on 18S have led to the recognition of three major lineages of nematodes: Dorylaimia (Clade I), Enoplia (Clade II), and Chromadoria, which consists of Spirurina (Clade III), Tylenchina (Clade IV), Rhabditina (Clade V), Plectida, Araeolaimida, Monhysterida, Desmodorida, and Chromadorida [17, 21, 22, 27–29]. Dorylaimia includes many free-living soil nematodes and plant parasites, but also vertebrate parasites such as *Trichinella,* whipworms, and *Dioctophyme*. Enoplia primarily consists of free-living aquatic nematodes, but also several lineages of soil nematodes and virus-transmitting plant pests (such as stubby root nematodes). Chromadoria includes a wide diversity of free-living aquatic nematodes but also familiar animal parasites (e.g. *Ascaris*, hookworms, and *Dirofilaria*), plant parasites (e.g. cyst and root knot nematodes), and the model organism *Caenorhabditis elegans*.

Enoplia has generally been thought to represent the sister group to all remaining nematodes [7, 30] because of the presence of presumably ancestral developmental features, which are common in other animal phyla but not seen in other lineages of nematodes thus far investigated. These include indeterminate development [31–33] and retention of the nuclear envelope in mature spermatozoa (other nematodes investigated to date have determinate development and spermatozoa that lose the nuclear envelope upon maturation [34]). As other metazoan lineages are thought to have marine origins, nematodes have traditionally been assumed to have evolved in the marine environment [24, 26, 35]. Thus the primarily marine habits of Enoplia, combined with their presumed ancestral developmental features, have led to them being viewed as the earliest-branching nematode lineage [7, 30]. On the other hand, De Ley and Blaxter [22] suggested the possibility of a terrestrial origin of nematodes with the sister group to all other nematodes being the taxon least represented in the marine environment, Dorylaimia. Ribosomal DNA-based studies have been unable to resolve the branching order among these deepest branches within Nematoda. Even studies focused on improved representation of diverse marine free-living nematodes [7, 36] failed to find resolution at the base of the nematode tree, suggesting that additional molecular markers are needed to resolve deep nematode phylogeny.

Recently, phylogenomic studies employing dozens to hundreds of nuclear protein-coding genes have addressed questions of nematode evolution, but taxon sampling in these studies has largely built on publicly available genome and transcriptome datasets [4, 37–41]. Until now, phylogenomic analyses of Nematoda have focused on parasitic taxa and model *Caenorhabditis* spp., with little or no representation of other free-living nematodes. For example, Blaxter and Koutsovoulos [40] and Koutsovoulos [41] curated the largest phylogenomic datasets for Nematoda to date but included only a single member of Enoplia in their studies. The latest comparative phylogenomic study focusing on parasitic worms included a handful of free-living nematodes (mostly model organisms), but no representatives of Enoplia or early branching Chromadoria [42]. Likewise, phylogenetic analyses based on mitochondrial genomes have never included representatives of Enoplia [43–46] because the mitochondrial genome has not yet been sequenced for any member of this clade.

Here, we have assembled the largest and most diverse phylogenomic dataset for Nematoda to date with expanded transcriptome representation for previously undersampled free-living nematode taxa. Leveraging this dataset, we re-examine relationships among early-branching clades and provide a robustly resolved and expanded phylogenetic framework for Nematoda.

## Results

Publicly available nematode and outgroup genomes and transcriptomes were supplemented with new transcriptomes from nine free-living nematodes, one nematomorph, and one kinorhynch for a total of 131 taxa sampled (Table 1, Additional file 2: Tables S1-S2). Building on an established phylogenomic data processing pipeline [47], we assembled two datasets using two different sequence selection strategies (see Methods). The first strategy used a strict orthology inference approach that refines initial orthology inference made by HaMStR [48] with PhyloTreePruner [49]. This strategy resulted in a dataset with 931 genes totalling 298,009 amino acids in length with 84.67% missing data. The second strategy employed SCaFoS [50] to select the best sequence for each taxon in the HaMStR output. The SCaFoS strategy resulted in a dataset with 1025 genes totalling 321,951 amino acids in length with 35.01% missing data.

**Table 1:** Accession numbers (NCBI BioProject), classification, locality, sequencing and assembly statistics, and HaMStR ortholog recovery (out of 1031) for newly generated transcriptomes

Accession number	Name	Locality	Reads	Bases sequenced	Transcripts	Min length	Max length	Mean length	HaMStR orthologs
	Nematoda								
PRJNA506144	*Anaplectus granulosus*, Plectidae, Plectida	Sweden, Stockholm city, soil (59.369243 N, 18.052779 E), ~50 individuals	8,455,308	963,909,283	83,260	201	5144	584	950
PRJNA506149	*Bathylaimus* sp., Tripyloididae, Enoplida	Sweden, Gullmarn fjord, 53 m depth, mud (58° 15.73′ N, 11°26.10′ E), 19 individuals	13,286,382	1,434,917,329	31,155	201	3743	499	461
PRJNA506150	*Euchromadora* sp., Chromadoridae, Chromadorida	Sweden, Gullmarn fjord, 53 m depth, mud (58° 15.73′ N, 11°26.10′ E), 39 individuals	13,097,922	1,414,602,399	75,347	201	8497	813	972
PRJNA506153	*Odontophora* sp., Axonolaimidae, Araeolaimida	Sweden, Skagerrak near Hållö, 14–17 m depth, sand (58° 20.35′ N, 11° 12.70′ E), ~60 individuals	8,413,939	754,420,192	41,153	201	3546	454	687
SRR8943407	Oncholaimidae gen. sp., Enoplida	Antarctica, Weddell Sea, 302 m depth (70° 53.52′ S, 11° 7.62′ W; PS96_163R), single individual	31,690,332	3,200,723,532	32,032	201	6985	576	565
PRJNA506154	*Pontonema* sp., Oncholaimidae, Enoplida	Sweden, Skagerrak near Hållö, 14–17 m depth, sand (58° 20.35′ N, 11° 12.70′ E), one individual	13,873,087	1,498,310,184	12,797	201	4863	594	347
PRJNA506156	*Symplocostoma* sp., Enchelidiidae, Enoplida	Sweden, Gullmarn fjord, 53 m depth, mud (58° 15.73′ N, 11°26.10′ E), 8 individuals	11,886,477	1,283,745,582	41,070	201	6051	556	378
SRR8943408	Thoracostomopsidae gen. sp., Enoplida	Antarctica, Weddell Sea, 302 m depth (70° 53.52′ S, 11° 7.62′ W; PS96_156R), single individual	29,345,050	2,963,850,050	16,794	201	8302	614	254
PRJNA506158	*Tobrilus* sp., Tobrilidae, Triplonchida	Sweden, Nacka, lake Källtorp, sandy sediment (59° 17.689′ N, 18° 09.608′ E), ~500 individuals	8,813,358	951,845,800	58,559	201	8807	774	999
	Nematomorpha								
SRR8943410	*Gordius* sp.	USA, Virginia, Rockbridge County, pond (37° 46.74667′ N, 79° 29.78167′ W), piece of midbody of single individual	35,319,538	3,567,273,338	39,939	201	14,897	1064	937
	Kinorhyncha								
SRR8943409	*Pycnophyes* sp.	Antarctica, Weddell Sea, 752.1 m depth (75° 51,14′ S, 032° 22,11′ W; PS96_1116R), single individual	27,441,206	2,771,561,806	85,908	201	10,886	547	563

**Fig. 1:**
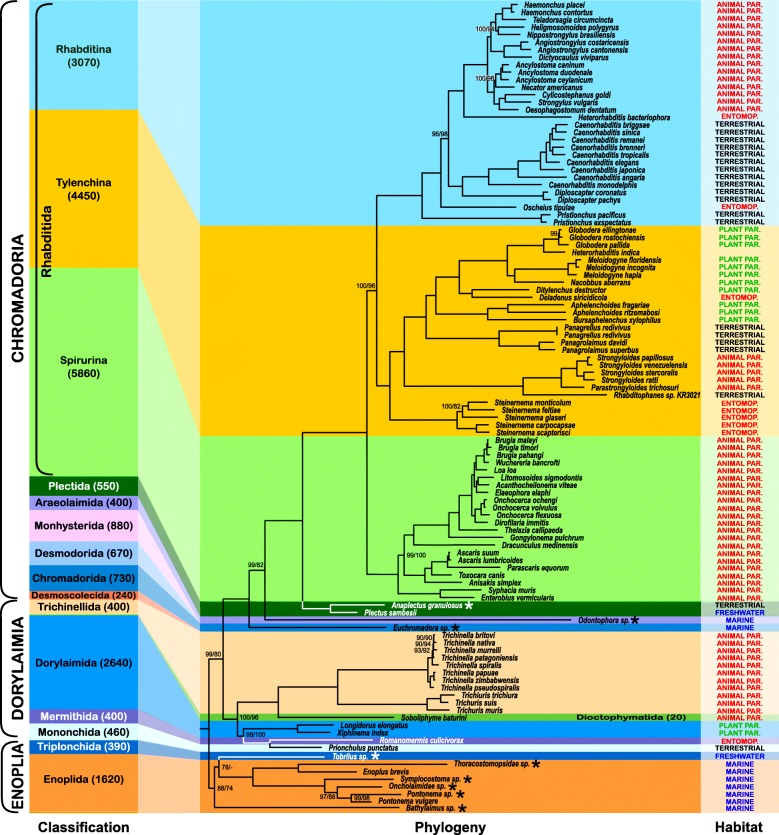
Phylogeny of Nematoda based on the IQ-TREE maximum likelihood analysis of the PhyloTreePruner dataset. “Classification” bar on the left side serves as a scale and represents the relative known taxonomic diversity of different taxa within Nematoda: the height of each colored bar is proportional to a number of known species (also given in the brackets after each taxon name), with the height of the entire multicolored background rectangle equal to 100% of known nematode diversity. IQ-TREE / RAxML bootstrap support values < 100% are shown. “Habitat” describes the lifestyle for each analysed species, such as animal parasitic (animal par.), plant parasitic (plant par.), entomopathogenic or entomoparasitic (entomop.), free-living freshwater (freshwater), terrestrial (terrestrial) and marine (marine). Newly generated transcriptomes are marked with an asterisk

Our results based on the matrix assembled with the more conservative PhyloTreePruner orthology inference strategy but with a higher proportion of missing data (Fig. 1) strongly support nematode monophyly (IQ-TREE / RAxML bootstrap support, bs = 100%/100%), and subsequent branching, with Enoplia being monophyletic (as previously recovered [7, 51]), and the sister clade to Dorylaimia and Chromadoria. Enoplida+Triplonchida was moderately supported (bs = 88%/74%). However, Enoplida was paraphyletic with respect to Triplonchida, a single representative of which, *Tobrilus* sp., was included as an ingroup. Dorylaimia and Chromadoria were recovered as sister taxa with strong support in the IQ-TREE analysis (bs = 99%) and moderate support in the RAxML analysis (bs = 80%).

Dorylaimia was strongly supported (bs = 100%/96%). This clade was primarily represented by members of the animal parasitic Trichinellida (*Trichinella* and *Trichuris*), which was also strongly supported as monophyletic (bs =100%/100%). Dorylaimida, which was represented by the virus-transmitting plant pests *Longidorus elongatus* and *Xiphinema index*, was also strongly supported as monophyletic (bs =100%/100%). Monophyly could not be tested for the remaining three orders represented by just one taxon each: Mononchida (represented by *Prionchulus punctatus*), Mermithida (represented by *Romanomermis culicivorax*), and Dioctophymatida (represented by *Soboliphyme baturini*). Mermithida was recovered as the sister to Mononchida with maximal support.

Chromadoria was strongly supported (bs =100%/100%) with the sole representative of Chromadorida (*Euchromadora* sp.) sister to a well-supported (bs = 99%/82%) clade of all other Chromadoria with *Odontophora* sp., the single representative of Araeolaimida, at the base. The two sampled representatives of Plectida (*Plectus sambesii* and *Anaplectus granulosus*) were recovered in a clade (bs =100%/100%) sister to Rhabditida. Rhabditida includes most described species of Chromadoria, and also most of the currently available transcriptomes and genomes. It received maximal support as did its three subclades: Spirurina, Tylenchina, and Rhabditina. Relationships within the major clades of Rhabditida were also consistently strongly supported. All genera for which monophyly was testable (i.e., those with more than one representative available for study), were recovered monophyletic, with the exception of *Heterorhabditis*. *Heterorhabditis bacteriophora* was strongly supported as sister to a clade composed of Trichostrongylidae and Ancylostomatidae within Rhabditina (as expected), while a species identified as *H. indica* was strongly supported as the sister taxon of *Globodera* spp. in Tylenchina.

Examination of the *Heterorhabditis indica* dataset [52] revealed that this organism was incorrectly identified or mislabelled – partial sequences of the nuclear ribosomal operon mined from the *H. indica* transcriptome assembly show high similarity to reference sequences from various species of the genera *Heterodera* and *Globodera* (Hoplolaimidae, Tylenchina), and not *Heterorhabditis* (Heterorhabditidae, Rhabditina). This is further confirmed by the results of our tree-based taxonomy assignment using the 18S rDNA gene fragment (Additional file 1: Figure S1). Unfortunately, these partial sequences mined from the transcriptome of *H. indica* are relatively short, one with only 588 bases of the 5′ end of 18S rDNA and the other with just 863 bases of the 5′ end of 28S rDNA. They do not contain enough phylogenetically informative sites to ensure species-level identification.

**Fig. 2:**
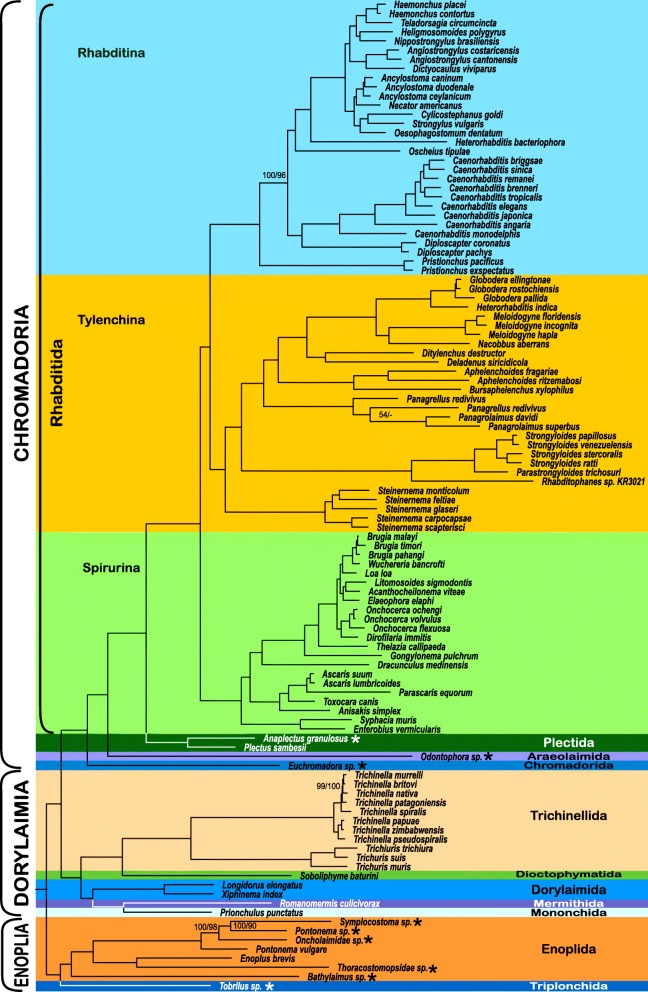
Phylogeny of Nematoda based on the IQ-TREE maximum likelihood analysis of the SCaFoS dataset. IQ-TREE / RAxML bootstrap support values < 100% are shown. Newly generated transcriptomes are marked with an asterisk

Because of the high amount of missing data (84.67%) in the dataset assembled using PhyloTreePruner, we also used a less conservative orthology inference approach that did not employ an additional tree-based orthology confirmation after initial HaMStR orthology inference. This resulted in a larger and much more complete dataset with 1026 genes totalling 321,951 amino acids in length with 64.99% matrix completeness. Analysis of this SCaFoS-based dataset resulted in a nearly identical branching order as that of the PhyloTreePruner-based dataset (Fig. 2). Whereas support for Enoplia was weak in the analysis of the PhyloTreePruner-based dataset, analysis of this dataset recovered Enoplia monophyletic and sister to the rest of Nematoda with maximal support. *Tobrilus* sp. (Triplonchida) was recovered sister to Enoplida with maximal support and *Bathylaimus* sp. was recovered sister to all other Enoplida with maximal support, which is in agreement with 18S rDNA-based analyses by van Megen et al. [53], Bik et al. [7], and Smythe [51]. Relationships within Dorylaimia were strongly supported and identical to the results based on the PhyloTreePruner dataset with the exception of relationships among *Trichinella nativa*, *T. britovi*, and *T. murrelli.* Likewise, relationships within Chromadoria were nearly identical; the one difference was placement of *Oscheius tipulae*, which was recovered sister to Rhabditomorpha sensu De Ley & Blaxter, 2004 [22] in the analysis of the PhyloTreePruner dataset and sister to Strongyloidea sensu De Ley & Blaxter, 2004 [22] in the analysis of the SCaFoS dataset.

**Fig. 3:**
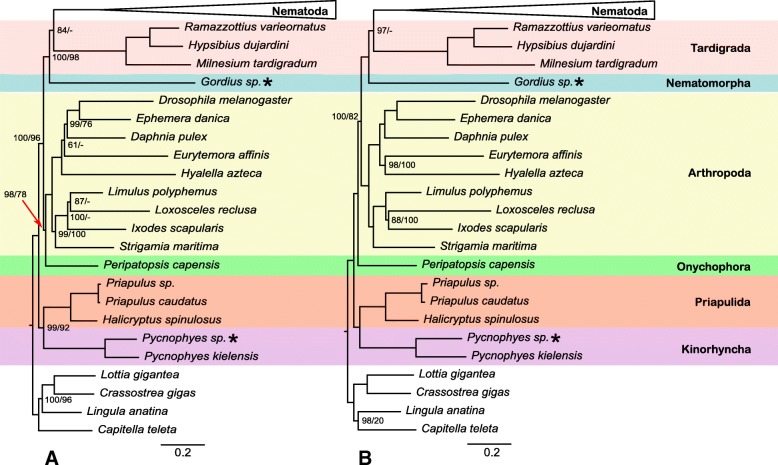
Phylogeny of outgroup taxa based on the IQ-TREE maximum likelihood analysis of the PhyloTreePruner (**a**) and SCaFoS (**b**) datasets. IQ-TREE / RAxML bootstrap support values < 100% are shown. Newly generated transcriptomes are marked with an asterisk

With respect to higher-level ecdysozoan (Fig. 3) relationships, both analyses recovered Scalidophora (represented by Priapulida + Kinorhyncha) monophyletic and sister to the rest of Ecdysozoa with strong support. IQ-TREE analysis of the PhyloTreePruner dataset recovered Onychophora sister to Arthropoda with strong support (bs = 98%) while the RAxML analysis had only moderate support for this placement (bs = 78%). However, analyses of the SCaFoS dataset recovered Onychophora sister to all non-scalidophoran ecdysozoans with similar levels of support (bs = 100%/82%). IQ-TREE analyses recovered Tardigrada sister to Nematoda with moderate to strong support (bs = 84–97%) whereas RAxML analyses recovered Tardigrada + Nematomorpha sister to Nematoda. This was strongly supported in the analysis of the SCaFoS dataset (bs = 100%) but weakly supported in the analysis of the PhyloTreePruner dataset (bs = 66%).

## Discussion

### Deep nematode phylogeny

Early evolution and diversification of nematodes has been a matter of much controversy (reviewed by [4, 15, 21, 22, 54]). Molecular phylogenetic studies have generally supported the existence of three major lineages and the monophyly of Chromadoria, but resolution of the deepest splits within Nematoda - relationships among Enoplia, Dorylaimia, and Chromadoria - has been recalcitrant. As in prior analyses based on 18S rDNA [7, 36, 53], analysis of our PhyloTreePruner-based dataset lacked support for relationships among these deepest branches in Nematoda. Enoplia received moderate support (bs = 88), while monophyly of Enoplida could not be established. Insufficient taxon sampling and limited matrix occupancy for Enoplia is, in our opinion, the prime issue to be considered and addressed in efforts to resolve relationships among these deep branches.

Our initial dataset assembly strategy employed PhyloTreePruner [49], which helps exclude paralogous sequences and contamination missed by the initial orthology inference approach. PhyloTreePruner examines single-gene trees and, if there are two or more sequences from a taxon that do not form a clade, the tree is pruned to the largest subclade in which all taxa are represented by just one sequence. Only the subset of sequences corresponding to that subtree is retained for concatenation and species tree reconstruction. Unfortunately, the PhyloTreePruner algorithm can result in the unnecessary exclusion of large numbers of sequences when even a single taxon has two or more sequences that do not form a clade in single-gene trees (Thálen and Kocot, unpublished data). Aside from paralogy, putative single-gene trees with two or more sequences from the same taxon that do not form a clade may also be caused by the presence of very short and/or mis-aligned contigs, low-quality contigs, or incorrect single gene trees. This problem is exacerbated as the number of sampled taxa increases (Thálen and Kocot, unpublished data).

Use of PhyloTreePruner with its strict orthology inference approach on this rather species-rich dataset resulted in exclusion of large subtrees worth of sequences for many of the orthogroups identified by HaMStR and a final concatenated dataset with just 15.33% matrix completeness. Because the HaMStR “model organisms” core ortholog set used in this study is known to consist of genes that are single copy across diverse metazoan phyla [48], paralogy is unlikely to be problematic with this dataset (although taxon-specific gene duplications are possible). Thus, we re-ran our pipeline using SCaFoS [50] to select sequences for concatenation. SCaFoS excludes highly divergent sequences (i.e., it is still able to exclude, non-nematode contamination) and selects the best sequence for each taxon based on average p-distance. As noted above, this resulted in a larger and much more complete dataset (64.99% matrix occupancy).

Despite substantial differences in matrix completeness, analysis of the SCaFoS-based matrix resulted in a very similar topology to that of the PhyloTreePruner-based matrix. Of significance, analysis of this more complete data matrix resulted in strong support for relationships among the major lineages of Nematoda, placing a monophyletic Enoplia sister to all other nematodes with maximal support, and supporting the monophyly of Enoplida. Our SCaFoS-based phylogeny supports the “traditional” view of early nematode evolution with Enoplia sister to the rest of Nematoda, a topology used as a basis for the long-standing yet poorly explored hypothesis that the phylum arose in the marine environment [22, 24, 26, 35]. The alternative hypothesis of the primarily terrestrial Dorylaimia as the sister to the rest of Nematoda [22], receives no support from either of our analyses.

**Fig. 4:**
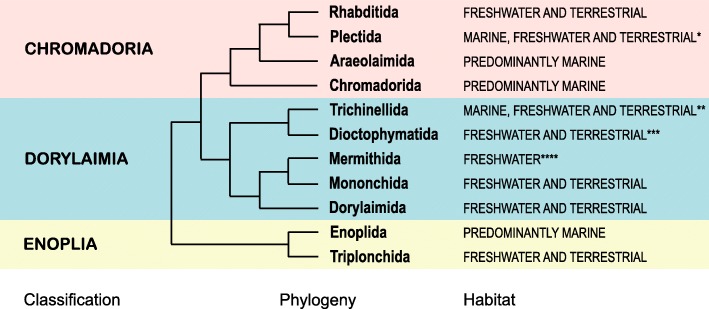
Simplified nematode phylogeny based on Fig. 2 indicating marine versus freshwater/terrestrial distribution for each order, considering the distribution of the majority of species. Notes: * includes equal number of marine, freshwater and terrestrial taxa, with molecular phylogenies suggesting terrestrial clades to be earlier (deeper); ** based on distribution of hosts, marine taxa may be of secondary origin; *** based on distribution of hosts; **** based on distribution of hosts and free-living stages

Placement of Enoplia as sister to the rest of Nematoda, however, does not deny the possibility of a terrestrial origin of Nematoda [22] as early-branching clades are equally represented by marine, freshwater and terrestrial taxa (Fig. 4). Enoplia splits into predominantly marine Enoplida and predominantly freshwater/terrestrial Triplonchida, while its sister clade (unnamed, containing the rest of Nematoda) consists of primarily marine Chromadoria and primarily freshwater/terrestrial Dorylaimia. A comprehensive hypothesis of nematode origin and early evolution must build on a greatly expanded phylogenomic dataset with better sampling of Enoplia and Dorylaimia and closely related phyla (Nematomorpha, Tardigrada, Priapulida, Kinorhyncha and Loricifera, Onychophora). This would better enable ancestral character state reconstruction analysis for Nematoda and Ecdysozoa as a whole.

### Relationships within major nematode clades

In terms of relationships within major nematode clades, our results are largely consistent with earlier studies based on the 18S rDNA gene [27, 30, 36, 53] and previous phylogenomic studies [40, 42]. One exception is the topology within Dorylaimia, which is somewhat different: 18S rDNA-based trees place Dorylaimida as the earliest branching clade [36, 53], although relationships among Mononchida, Mermithida, Trichinellida and Dioctophymatida vary. Our results place a clade containing Dorylaimida, Mermithida and Mononchida sister to a clade with Dioctophymatida and Trichinellida. Our recovery of Mermithida as the sister taxon of Mononchida is in agreement with 18S rDNA based phylogenetic studies (e.g. [27], but in discordance with morphology-based theories, which suggest closer affinities between Mermithida and Dorylaimida [55, 56] or Mermithida and Trichinellida (=Trichocephalida) [57]. Another exception is in the branching pattern of Rhabditida: our analysis places Spirurina as a sister to Tylenchina + Rhabditina (in full agreement with all 18S rDNA-based and most phylogenomic studies), while [42] recovered Tylenchina as a sister to Rhabditina + Spirurina, albeit with relatively low bootstrap support.

### “Minor” problems in nematode phylogeny

Early radiation within the phylum Nematoda is the most challenging problem but not the only one in the systematics of this group of animals. There are a number of “orphaned” nematode taxa for which phylogenetic affinities and thus placement in the classification remain unclear. Such are the phylogenetic relationships of nematode families Teratocephalidae [22], Chambersiellidae [58], Brevibuccidae [22], Myolaimidae [59], Aegialoalaimidae [60], Cyartonematidae [61], Aulolaimidae [62, 63], Paramicrolaimidae [60, 64], Haliplectidae [60], Richtersiidae [65], Rhabdodemaniidae [51, 66], Thalassogeneridae [67], suborder Ceramonematina [60] and orders Benthimermithida [68, 69], Marimermithida [70] and Rhaptothyreida [71]. They often possess unusual morphologies [59, 63, 64] or are highly specialized parasites [69, 70], and have no clear place in morphology-based classifications.

Acquisition of transcriptome or genome data from the understudied taxa is needed in order to resolve these “minor” phylogenetic issues that could not be clarified in phylogenetic studies based on rDNA loci or morphology, which have provided contradictory results depending on the data or methodology used. Besides finally achieving stable classification, many of these taxa are important for understanding of morphological character evolution, transitions between marine and terrestrial lifestyles, and evolution of symbiosis in the marine environment.

### Phylogeny of Ecdysozoa

Although taxon sampling of the present study focused on Nematoda, we aimed to broadly sample relevant outgroups using only high-quality, publicly available data plus new transcriptomes from a nematomorph and a kinorhynch. Relationships among ecdysozoan phyla have varied somewhat dramatically among studies (reviewed by [72]), prompting numerous conflicting phylogenetic hypotheses. Our results find no support for some traditionally hypothesized groups including Nematoida (Nematoda + Nematomorpha), Panarthropoda (Arthropoda, Onychophora, and Tardigrada), or Cycloneuralia (Scalidophora + Nematoida). Interestingly, we recover Tardigrada as the sister taxon of Nematoda. A close relationship of Tardigrada to Nematoda has been recovered in other recent phylogenomic studies [73–77], but data from representatives of Nematomorpha have been limited. Interestingly, the PhyloTreePruner-based analysis recovers the traditionally hypothesized placement of Onychophora as the sister taxon of Arthropoda with strong support (bs = 98) but in the SCaFoS-based analysis, it is recovered as the sister taxon of a clade of all other non-scalidophoran ecdysozoans with maximal support. The limited taxon sampling for key ecdysozoan clades (e.g., just one onychohoran, one nematomorph, no heterotardigrades, no loriciferans, etc.) further demonstrates the need for high-quality genomic and transcriptomic resources from this part of the animal tree.

### Expand sampling of free-living nematodes to learn more about parasites

The origin and evolution of animal parasitic nematodes from their free-living ancestors has been an active area of research for 80 years [78–83]. Two simplified scenarios describe evolutionary pre-adaptations and morpho-physiological changes leading towards parasitism via commensalism in aquatic environments [84, 85] and via a saprobiontic lifestyle in terrestrial environments [80, 86, 87]. We are just beginning to understand the genomic changes involved in these processes [42, 88]. Furthermore, many other important questions about parasite biology remain unanswered, such as how parasites locate and invade hosts, suppress host immune response, acquire nutrients, etc. [40].

Comprehensive understanding of morphological, ecological, behavioral and genomic adaptations involved in the evolution of a parasitic lifestyle can not be achieved without thorough comparison between parasites and their close, free-living relatives [19, 40, 89]. One of the complications, however, is that animal parasitic nematodes evolved independently at least 18 times [90], if not more [40, 86], and one cannot expect the same underlying mechanism to be behind these numerous independent events. Moreover, the majority of animal parasitic clades have no identified, closely related free-living taxon suitable for comparative analysis [19]. These include all parasites from the subclass Dorylaimia and the most diverse and economically important Spirurina. Even the closest relative of such a well-researched taxon as the entomopathogenic genus *Steinernema* remains unclear [58, 91]. Thus, further expanding sampling of free-living nematodes in phylogenomic studies will be an integral part of any future research aiming to understand the evolution of parasitism – it will help elucidate sister-group relationships of those parasitic taxa for which the closest free-living relatives are yet unidentified and provide much needed comparative data for identification of parasitism-related genetic modifications.

With 97 published and nine new nematode genomes and transcriptomes, our phylogenetic analyses, which are by far the most comprehensive to date, cover less than 0.5% of the approximately 23,000 valid nematode species [92]. For comparison, the latest phylum-wide 18S rDNA-based phylogeny [53] included 1215 sequences or just about 5% of the known diversity. Of the 108 nematode species included in our analyses, 80 belong to Rhabditida – a clade with over 13,400 known species including most economically and medically important parasites as well as the model species *Caenorhabditis elegans* and satellite model *Pristionchus pacificus*. Of the Rhabditida species included in our analyses, 50 are parasites of animals, 12 are plant parasitic, and the remaining 18 are thought to be free-living inhabitants of soil or saprophytic communities (although some are phoretically associated with invertebrates). The next largest set of species, 11 in number, represent exclusively the parasitic order Trichinellida (with about 400 known species). The remainder of the phylum, consisting of 17 orders and including free-living (in particular almost all known marine species), plant- and animal parasitic nematodes (with about 9200 species in total), is unevenly represented in our analysis: nine orders are represented by 17 species, while eight orders are not included at all. Out of 108 species included in this phylogenetic analysis, 63 are animal parasitic and 14 are associated with plants, while only 31 are free-living, of which 22 are fresh water and soil inhabitants and only nine are marine. Thus, vast habitat diversity, and the morphological and molecular adaptations that allow nematodes to live in those environments, remains unrepresented in transcriptome-based phylogenies.

### Recommended sampling strategies

Three possible sampling strategies to increase and diversify nematode genomic and transcriptomic datasets can be suggested, depending on the research goals. Those researchers who are interested solely in the origin and early evolution of animal parasitism can find interesting models among free-living Enoplida [93], Chromadorida [94], Monhysterida [95, 96] and Plectida [97–99] – species with parasitic lifestyles but with morphology retaining many features of their close, free-living relatives. Phylogenetic analysis and subsequent ancestral character state reconstruction would elucidate features of free-living ancestors of parasites and generate new hypotheses regarding the evolution of parasitism. Secondly, studies aimed at improving general nematode phylogeny and classification must focus on the species described above in the “Minor” problems in nematode phylogeny and taxa to which they were once believed to be related to. Finally, large taxonomic categories currently represented by single or few genomes/transcriptomes (Triplonchida, Mononchida, Dorylaimida, etc) also deserve attention, and further sampling of those taxa would elucidate relationships in those clades and likely spur research into yet more unanswered questions.

## Conclusion

This study represents the largest phylogenomic analysis of nematodes to date, and furthers our understanding of nematode relationships. We have also, however, revealed how poorly sampled the current dataset is relative to the tremendous diversity of nematodes on Earth. Sequencing and re-sequencing of more species and broad scale comparative studies can also reveal and correct misidentified or mislabelled datasets (the case of *Heterorhabditis indica*). Transcriptome sequencing of nematodes is still strongly biased toward parasitic and “model” taxa, particularly those in the Rhabditida, neglecting the free-living clades that hold the key to the origins of the phylum. Our understanding of nematode early evolution and various pathways towards parasitism will be improved only by broader sampling and sequencing of free-living taxa.

## Methods

Nematodes and the kinorhynch were collected and isolated following standard protocols for sampling meiofauna [100]. Immediately after isolation, live specimens of *Anaplectus granulosus, Euchromadora* sp., *Symplocostoma* sp., and *Tobrilus* sp. were frozen in 100 μL of nuclease-free water at − 70 °C. *Bathylaimus* sp., *Gordius* sp., *Odontophora* sp., Oncholaimidae sp., *Pontonema* sp., *Pycnophyes* sp., and Thoracostomopsidae sp. were preserved in RNA*later* and stored at − 20 °C.

Total RNA was extracted from all samples but *Gordius* sp. using the Ambion RNAqueous-Micro Kit. For *Anaplectus granulosus, Euchromadora* sp. and *Tobrilus* sp., 1000 μL of lysis solution was added directly to the original sample (nematodes in 100 μl of nuclease-free water), while individual specimens of the remaining nematodes and the kinorhynch were manually transferred from RNAlater or nuclease-free water to lysis solution. Subsequent steps of RNA extraction and DNAse treatment followed the manufacturer’s protocol. RNA was extracted from the nematomorph *Gordius* sp. using the Omega Bio-Tek EZNA Mollusc RNA kit using a rotor-stator homogenizer for homogenization and on-column DNAse treatment.

For *Anaplectus granulosus*, *Bathylaimus* sp., *Euchromadora* sp., *Odontophora* sp., *Pontonema* sp., *Symplocostoma* sp. and *Tobrilus* sp., library preparation and cDNA synthesis was performed using the Clontech SMARTer PCR cDNA Synthesis Kit following manufacturer’s instructions. Resulting double-stranded cDNA was purified using the QIAquick PCR Purification Kit. Concentration of double-stranded cDNA was measured using Qubit dsDNA HS Assay Kit and Qubit 3.0 Fluorometer. Final library preparation and transcriptome sequencing were performed at the Swedish National Genomics Infrastructure in Stockholm, Sweden using the Illumina TruSeq PCR-free protocol and an Illumina HiSeq 2500 in high-output mode with V4 2 X 125 bp paired-end reads.

For Oncholaimidae sp. and Thoracostomopsidae sp., total RNA (not quantified; < 1 ng) was sent to Macrogen Inc. (Seoul, South Korea) for cDNA library preparation with the SMARTer low input RNA kit and sequencing using on the Illumina HiSeq 2500 using HiSeq SBS V4 with 2 X 100 bp paired-end reads. For *Gordius* sp., total RNA (1 μg) was sent to Macrogen for Illumina TruSeq RNA library preparation and sequencing using the Illumina HiSeq 2500 using HiSeq SBS V4 with 2 X 100 bp paired-end reads.

Dataset assembly and analysis followed the approach of Kocot et al. [47]. Publicly available genomic data [101, 102] were downloaded as predicted proteins if available (Additional file 2: Table S1). Transcriptome dataset of *Plectus sambesii* was provided by Dr. Philipp Schiffer (CLOE, University College London, UK) and Dr. Christopher Kraus (Zoological Institute, Universität zu Köln, Germany), while transcriptome of *Pontonema vulgare* was provided by Dr. Andreas Hejnol (Sars International Centre for Marine Molecular Biology, University of Bergen, Norway). Otherwise, predicted transcripts from genomes or assembled transcriptomes were downloaded when possible. After demultiplexing, raw reads for *Anaplectus granulosus*, *Bathylaimus* sp., *Euchromadora* sp., *Odontophora* sp., *Pontonema* sp., *Symplocostoma* sp. and *Tobrilus* sp. were filtered using AfterQC [103] and assembled with Trinity [104] installations available on public Galaxy [105] servers at usegalaxy.org (Center for Comparative Genomics and Bioinformatics at Penn State, the Department of Biology and at Johns Hopkins University and the Computational Biology Program at Oregon Health & Science University) or galaxy.ncgas-trinity.indiana.edu (National Center for Genome Analysis Support, Pervasive Technology Institute at Indiana University). *Pycnophyes* sp., *Gordius* sp., Oncholaimidae sp. and Thoracostomopsidae sp. as well as publicly available transcriptomes available only as raw reads were quality filtered, adapter-trimmed, and assembled using Trinity 2.2.0 with the --trimmomatic and --normalize_reads flags [104] on the University of Alabama UAHPC cluster. Transcripts were translated with TransDecoder 2.0.0 or 2.0.1 [106] using the UniProt SwissProt database (accessed on September 20th, 2016; The Uniprot Consortium 2014) and PFAM (Pfam-A.hmm) version 27 [107].

For orthology inference, HaMStR 13 [48] was used with the “model organisms” core-ortholog set. Translated transcripts for all taxa except *Caenorhabditis elegans* were searched against the 1031 profile hidden Markov models (pHMMs) using the “-central” flag and otherwise with the default options. Sequences matching a pHMM were compared to the proteome of *Caenorhabditis elegans* using BLASTP with the default search settings of HaMStR. If the *Caenorhabditis elegans* amino acid sequence contributing to the pHMM was the best BLASTP hit in each of these back-BLASTs, the sequence was then assigned to that putative orthology group (simply referred to as “gene” henceforth). Redundant sequences that were identical (including partial sequences that were identical at least where they overlapped) were then removed with UniqHaplo (http://raven.wrrb.uaf.edu/~ntakebay/teaching/programming/perl-scripts/uniqHaplo.pl), leaving only unique sequences for each taxon. Each gene was then aligned with MAFFT 7.215 using the automatic alignment strategy with a “maxiterate” value of 1000 [108]. Alignments were then trimmed with BMGE (−g 0.5) to remove ambiguously aligned regions and any alignments shorter than 50 bp were deleted. Sequences that did not overlap with all other sequences in the alignment by at least 20 amino acids were deleted, starting with the shortest sequences not meeting this criterion. This step was necessary for downstream single-gene tree reconstruction. Finally, genes sampled for fewer than 10 taxa after these steps were discarded.

In some cases, a taxon was represented in an alignment by two or more sequences (splice variants, lineage-specific gene duplications [=inparalogs], undetected paralogs, or exogenous contamination). To screen for evidence of paralogy or contamination and select just one sequence for each taxon, an approximately maximum likelihood tree was inferred for each remaining alignment using FastTree 2 [109] using the -slow and -gamma options. PhyloTreePruner [49] was then employed to use a tree-based approach to screen each single-gene alignment for evidence of paralogy or contamination. First, nodes with support values below 0.95 were collapsed into polytomies. Next, the maximally inclusive subtree was selected where each taxon was represented by no more than one sequence or, in cases where more than one sequence was present for any taxon, all sequences from that taxon formed a clade or were part of the same polytomy. Putative paralogs and contaminants (sequences falling outside of this maximally inclusive subtree) were then deleted from the input alignment. In cases where multiple sequences from the same taxon formed a clade or were part of the same polytomy, all sequences except the longest were deleted. Concatenation of remaining sequences to assemble the data matrix henceforth referred to as the “original full dataset” was performed using FASconCAT-G [110].

Because PhyloTreePruner can result in the unnecessary exclusion of large numbers of sequences when even a single taxon has unstable or contaminant sequences, we also ran our pipeline using SCaFoS [50] instead of PhyloTreePruner. The default settings were used to exclude highly divergent sequences and select the best sequence for each taxon based on average p-distance to all other sequences in the alignment.

Maximum likelihood (ML) analyses were conducted in RAxML 8.2.8 [111] and IQ-TREE [112]. Because of the very large number of taxa in our matrices, for the RAxML analyses, data matrices were partitioned by gene but the PROTGAMMALG model was specified for all partitions rather than empirically inferring the best-fitting model for each partition. A preliminary run of PartitionFinder 2 [113] found that the LG model was the best fit for the vast majority of partitions. The tree with the best likelihood score after 10 random addition sequence replicates was retained and nodal support was assessed with rapid bootstrapping with the number of replicates determined by the autoMRE criterion. IQ-TREE analyses were performed using IQ-TREE 1.5.5 with the site heterogeneous PMSF model [63] (−m LG + C20 + G + F) with the RAxML bipartitions tree provided as the required guide tree (−ft). Nodal support was assessed with 1000 rapid bootstraps (−bb 1000).

Several taxonomy assignment approaches were used to identify the transcriptome of *Heterorhabditis indica*. At first, transcriptome database for *Heterorhabditis indica* available at http://insilico.iari.res.in/hindica/was mined for possible ribosomal DNA sequences using built-in BLAST search and 18S rDNA sequence of *Plectus aquatilis* (chosen to be equally distantly related from both *Heterorhabditis* and *Heterodera*) as a target. Four recovered transcripts were then compared with the publicly available sequences from the nucleotide collection of NCBI GenBank using blastn suite (alignment-based taxonomy assignment approach, see review in [114]). One of the recovered transcripts (labelled as Locus_123_Transcript_1/1) showed high similarity (> 99% identity, E-value = 0) to several 18S sequences from different species of the genera *Heterodera* and *Globodera*, with *Heterodera glycines* (GenBank acc. Number AY043247) having the highest identity score, albeit with partial overlap. The other two transcripts (labelled as Locus_90_Transcript_1/2 and Locus_90_Transcript_2/2 respectively) also showed high similarity (99% identity, E-value = 0) to several sequences from different species of the genera *Heterodera* and *Globodera*, partially overlapping various reference sequences that may include partial 18S, ITS1, 5.8S, ITS2 and partial 28S, with *Heterodera cajani* (GenBank acc. Number AY274389) having the highest identity score. Similar results were obtained by mining the transcriptome assembly downloaded directly from GenBank.

The longest section of 18S rDNA sequence mined from the *Heterorhabditis indica* transcriptome database (588 base long partial 5′ section from the Locus_123_Transcript_1/1) was then used in tree-based taxonomy assignment approach (see review in [115]) to double-check the results of alignment-based taxonomy assignment. This section was added to a selection of 18S rDNA sequences downloaded from SILVA database [116] and representing all major clades of Rhabditida, including all available near-full length sequences for identified species from the genera *Heterorhabditis, Heterodera* and *Globodera.* The alignment was created using MUSCLE at https://www.ebi.ac.uk/Tools/msa/muscle/ under default settings and trimmed to a size of a fragment from the *Heterorhabditis indica* transcriptome. A phylogenetic tree was inferred using RAxML-HPC2 under default settings with 1000 bootstrap replicates.

## Additional files

Additional file 1: **Figure S1.** Phylogenetic position of 18S rDNA fragment (contig Locus_123_Transcript_1/1) extracted from *Heterorhabditis indica* transcriptome dataset (http://insilico.iari.res.in/hindica/). (PDF 46 kb)

Additional file 2: **Table S1.** Names, classification (family and order), accession number (BioProject) or download link, and source/citation for publicly available genomes and transcriptomes of nematodes used in phylogenetic analysis. Classification is based on [1] with modifications [2, 3]. **Table S2.** Names and origin data or reference for publicly available genomes, transcriptomes or proteomes of non-nematode (outgroup) taxa. (DOCX 22 kb)

## Data Availability

The datasets generated and analysed during the current study including transcriptome assemblies are available via FigShare: https://figshare.com/s/4c8e501714dbd5be1be8

## Abbreviations

bs: Bootstrap support
ML: Maximum likelihood

## References

[1] Hugot JP, Baujard P, Morand S (2001). Biodiversity in helminths and nematodes as a field of study: an overview. Nematology..

[2] Baldwin JG, Nadler SA, Wall DH, Raven P, Williams T (2000). Nematodes: pervading the earth and linking all life. Nature and human society: the quest for a sustainable world: proceedings of the 1997 forum on biodiversity.

[3] Appeltans W, Ahyong ST, Anderson G, Angel MV, Artois T, Bailly N (2012). The magnitude of global marine species diversity. Curr Biol.

[4] Blaxter M, Koutsovoulos G, Jones M, Kumar S, Elsworth B. Phylogenomics of Nematoda. In: Cotton JA, Hughes J, Olson P, editors. Next-generation systematics. Cambridge: Cambridge University Press; 2016. p. 62–83. https://books.google.com/books?hl=en&lr=&id=C3JNDAAAQBAJ&oi=fnd&pg=PR9&dq=Blaxter+M,+Koutsovoulos+G,+Jones+M,+Kumar+S,+Elsworth+B.+Phylogenomics+of+Nematoda.+In:+Cotton+JA,+Hughes+J,+Olson+P,+editors.+Nextgeneration+systematics:+Cambridge+University+Press%3B+2016.+p.+62%E2%80%9383.&ots=a4ebTDtW03&sig=J4P4jtLKs0kRpj-B_MiyGBwpdOQ#v=onepage&q&f=false.

[5] Warwick RM, Rice R (1979). Ecological and metabolic studies on free living nematodes from an estuarine mudflat. Estuar Coast Shelf Sci.

[6] Lambshead PJD, Schalk P, Levin S (2001). Overview of marine invertebrate biodiversity. Encyclopedia of biodiversity.

[7] Bik HM, Lambshead PJD, Thomas WK, Lunt DH (2010). Moving towards a complete molecular framework of the Nematoda: a focus on the Enoplida and early-branching clades. BMC Evol Biol.

[8] Fonseca VG, Carvalho GR, Sung W, Johnson HF, Power DM, Neill SP (2010). Second-generation environmental sequencing unmasks marine metazoan biodiversity. Nat Commun.

[9] Coull BC (1990). Are members of the meiofauna food for higher trophic levels?. Trans Am Microsc Soc.

[10] Lambshead P, Brown C, Ferrero T, Hawkins L, Smith C, Mitchell N (2003). Biodiversity of nematode assemblages from the region of the clarion-Clipperton fracture zone, an area of commercial mining interest. BMC Ecol.

[11] Bert W, Manhout J, Van Colen C, Borgonie G, Decraemer W (2009). Nematode assemblages in a nature reserve with historical pollution. Belg J Zool.

[12] Gingold R, Moens T, Rocha-Olivares A (2013). Assessing the response of nematode communities to climate change-driven warming: a microcosm experiment. PLoS One.

[13] Andrus P., Rae R. (2018). Development of Phasmarhabditis hermaphrodita (and members of the Phasmarhabditis genus) as new genetic model nematodes to study the genetic basis of parasitism. Journal of Helminthology.

[14] Derycke S, Backeljau T, Moens T (2013). Dispersal and gene flow in free-living marine nematodes. Front Zool.

[15] Chitwood BG, Chitwood MB (1974). Introduction to nematology, revised edition.

[16] Maggenti A (1981). General Nematology.

[17] De Ley P. A quick tour of nematode diversity and the backbone of nematode phylogeny. In The *C. elegans* Research Community, editor. WormBook. (January 25, 2006). 10.1895/wormbook.1.41.1. 10.1895/wormbook.1.41.1 PMC4781170 18050465

[18] Copley JTP, Flint HC, Ferrero TJ, Van Dover CL (2007). Diversity of melofauna and free-living nematodes in hydrothermal vent mussel beds on the northern and southern east pacific rise. J Mar Biol Assoc UK.

[19] Viney M (2017). How can we understand the genomic basis of nematode parasitism?. Trends Parasitol.

[20] Dorris M, De Ley P, Blaxter M (1999). Molecular analysis of nematode diversity and the evolution of parasitism. Parasitol Today.

[21] De Ley P, Blaxter ML, Lee D (2002). Systematic position and phylogeny. The biology of nematodes.

[22] De Ley P, Blaxter ML (2004). A new system for Nematoda: combining morphological characters with molecular trees, and translating clades into ranks and taxa. Nematology Monographs and Perspectives.

[23] Chitwood BG (1958). The designation of official names for higher taxa of invertebrates. Bull Zool Nomencl.

[24] Maggenti AR, Dougherty E, Brown Z, Hanson E, Hartman W (1963). Comparative morphology in nemic phylogeny. The lower Metazoa, comparative biology and phylogeny.

[25] Andrássy I (1976). Evolution as a basis for the systematization of nematodes.

[26] Malakhov VV (1994). Nematodes: structure, development, classification, and phylogeny.

[27] Blaxter ML, De Ley P, Garey JR, Liu LX, Scheldeman P, Vierstraete A (1998). A molecular evolutionary framework for the phylum Nematoda. Nature..

[28] Smythe A, Sanderson M, Nadler S (2006). Nematode small subunit phylogeny correlates with alignment parameters. Syst Biol.

[29] Donn S, Neilson R, Griffiths BS, Daniell TJ (2011). Greater coverage of the phylum Nematoda in SSU rDNA studies. Biol Fertil Soils.

[30] Holterman M, van der Wurff A, van den Elsen S, van Megen H, Bongers T, Holovachov O (2006). Phylum-wide analysis of SSU rDNA reveals deep phylogenetic relationships among nematodes and accelerated evolution towards crown clades. Mol Biol Evol.

[31] Hope I, Lee D (2002). Embryology, developmental biology and the genome. The biology of nematodes.

[32] Schierenberg E (2005). Unusual cleavage and gastrulation in a freshwater nematode: developmental and phylogenetic implications. Dev Genes Evol.

[33] Schulze J, Schierenberg E (2011). Evolution of embryonic development in nematodes. EvoDevo..

[34] Justine J, Lee D (2002). Male and female gametes and fertilisation. The biology of nematodes.

[35] Paramonov AA. Principles of phytonematology Vol. 1. The origin of nematodes. Ecological and morphological characteristics of phytonematodes. General principles of taxonomy. Izdatelstvo Akademii Nauk SSSR; 1964.

[36] Meldal BHM, Debenham NJ, De Ley P, De Ley IT, Vanfleteren JR, Vierstraete A (2007). An improved molecular phylogeny of the Nematoda with special emphasis on marine taxa. Mol Phylogenet Evol.

[37] Parkinson J, Mitreva M, Whitton C, Thomson M, Daub J, Martin J (2004). A transcriptomic analysis of the phylum Nematoda. Nat Genet.

[38] Wasmuth J, Schmid R, Hedley A, Blaxter M (2008). On the extent and origins of genic novelty in the phylum Nematoda. PLoS Negl Trop Dis.

[39] Blaxter M, Kumar S, Kaur G, Koutsovoulos G, Elsworth B (2012). Genomics and transcriptomics across the diversity of the Nematoda. Parasite Immunol.

[40] Blaxter M, Koutsovoulos G (2015). The evolution of parasitism in Nematoda. Parasitology..

[41] Koutsovoulos G. Reconstructing the phylogenetic relationships of nematodes using draft genomes and transcriptomes. PhD [dissertation]. Edinburgh, Scotland: University of Edinburgh; 2015.

[42] International Helminth Genomes Consortium. Comparative genomics of the major parasitic worms. Nat Genet. 2018. 10.1038/s41588-018-0262-1. 10.1038/s41588-018-0262-1 PMC6349046 30397333

[43] Kang S, Sultana T, Eom KS, Park YC, Soonthornpong N, Nadler SA (2009). The mitochondrial genome sequence of *Enterobius vermicularis* (Nematoda: Oxyurida) – an idiosyncratic gene order and phylogenetic information for chromadorean nematodes. Gene..

[44] Kim J, Lee S-H, Gazi M, Kim T, Jung D, Chun J-Y (2015). Mitochondrial genomes advance phylogenetic hypotheses for Tylenchina (Nematoda: Chromadorea). Zool Scr.

[45] Kim J, Kern E, Kim T, Sim M, Kim J, Kim Y (2017). Phylogenetic analysis of two *Plectus* mitochondrial genomes (Nematoda: Plectida) supports a sister group relationship between Plectida and Rhabditida within Chromadorea. Mol Phylogenet Evol.

[46] Park J-K, Sultana T, Lee S-H, Kang S, Kim HK, Min G-S (2011). Monophyly of clade III nematodes is not supported by phylogenetic analysis of complete mitochondrial genome sequences. BMC Genomics.

[47] Kocot KM, Tassia MG, Halanych KM, Swalla BJ (2018). Phylogenomics offers resolution of major tunicate relationships. Mol Phylogenet Evol.

[48] Ebersberger I, Strauss S, vo Haeseler A (2009). HaMStR: profile hidden markov model based search for orthologs in ESTs. BMC Evol Biol.

[49] Kocot KM, Citarella MR, Moroz LL, Halanych KM (2013). PhyloTreePruner: a phylogenetic tree-based approach for selection of orthologous sequences for Phylogenomics. Evol Bioinformatics Online.

[50] Roure B, Rodriguez-Ezpeleta N, Philippe H (2007). SCaFoS: a tool for selection, concatenation and fusion of sequences for phylogenomics. BMC Evol Biol.

[51] Smythe AB (2015). Evolution of feeding structures in the marine nematode order Enoplida. Integr Comp Biol.

[52] Somvanshi VS, Gahoi S, Banakar P, Thakur PK, Kumar M (2016). A transcriptomic insight into the infective juvenile stage of the insect parasitic nematode, *Heterorhabditis indica*. BMC Genomics.

[53] van Megen H, van den Elsen S, Holterman M, Karssen G, Mooyman P, Bongers T (2009). A phylogenetic tree of nematodes based on about 1200 full-length small subunit ribosomal DNA sequences. Nematology..

[54] Coomans A (2002). Present status and future of nematode systematics. Nematology..

[55] Steiner G (1917). Über die Verwandtschaftsverhältnisse und die systematische Stellung der Mermithiden. Zool Anz.

[56] Poinar GO, Hess R (1974). Structure of the pre-parasitic juveniles of *Filipjevimermis leipsandra* and some other Mermithidae (Nematodea). Nematologica..

[57] Rubtsov IA. Mermithids. Origin, biology, distribution. Nauka; 1977.

[58] Holovachov O, Camp L, Nadler SA (2015). Sensitivity of ribosomal RNA character sampling in the phylogeny of Rhabditida. J Nematol.

[59] Slos D, Couvreur M, Bert W (2018). Description of *Myolaimus mycophilus* Slos & Bert sp. n. (Rhabditida: Myolaimidae). Nematology..

[60] Holovachov O, Schmidt-Raesa A (2014). Chapter 7.16: order Plectida Gadea, 1973. Handbook of zoology. Gastrotricha, Cycloneuralia and Gnathifera. Volume 2: Nematoda.

[61] Tchesunov AV (1994). On the morphology and systematic position of the family Meyliidae (Nematoda: Chromadoria). Nematologica..

[62] Holovachov O, Bostrőm S, Susulovsky A (2007). Description of *Aulolaimus multipapillatus* sp. n. and *A. nannocephalus* Andrássy, 1972 with notes on taxonomy and phylogeny of the genus (Nematoda: Aulolaimidae). Nematology..

[63] Abolafia J, Peña-Santiago R (2018). Morphology, taxonomy and phylogeny of the enigmatic genus Aulolaimus de man, 1880 (Nematoda, Aulolaimidae). Zool Anz.

[64] Leduc D, Verdon V, Zhao Z (2018). Phylogenetic position of the Paramicrolaimidae, description of a new *Paramicrolaimus* species and erection of a new order to accommodate the Microlaimoidea (Nematoda: Chromadorea). Zool J Linnean Soc.

[65] Tchesunov AV, Schmidt-Raesa A (2014). Chapter 7.13: Order Desmodorida De Coninck, 1965. Handbook of zoology. Gastrotricha, Cycloneuralia and Gnathifera. Volume 2: Nematoda.

[66] Holovachov O, Shoshin A, Schmidt-Raesa A (2014). Chapter 7.4: order Triplonchida cobb, 1919. Handbook of zoology. Gastrotricha, Cycloneuralia and Gnathifera. Volume 2: Nematoda.

[67] Smol N, Muthumbi A, Sharma J, Schmidt-Raesa A (2014). Chapter 7.3: Order Enoplida. Handbook of zoology. Gastrotricha, Cycloneuralia and Gnathifera. Volume 2: Nematoda.

[68] Holovachov O, Rodrigues CF, Zbinden M, Duperron S (2013). *Trophomera conchicola* sp. n. (Nematoda: Benthimermithidae) from chemosymbiotic bivalves *Idas modiolaeiformis* and *Lucionoma kazani* (Mollusca: Mytilidae and Lucinidae) in eastern Mediterranean. Russ J Nematol.

[69] Miljutin DM, Schmidt-Raesa A (2014). Chapter 7.1: order Benthimermithida Tchesunov, 1995. Handbook of zoology. Gastrotricha, Cycloneuralia and Gnathifera. Volume 2: Nematoda.

[70] Miljutin DM, Schmidt-Raesa A (2014). Chapter 7.10: order Marimermithida Rubtzov 1980, emend. Tchesunov, 1995. Handbook of zoology. Gastrotricha, Cycloneuralia and Gnathifera. Volume 2: Nematoda.

[71] Leduc D, Zhao Z, Verdon V, Xu Y (2018). Phylogenetic position of the enigmatic deep-sea nematode order Rhaptothyreida: a molecular analysis. Mol Phyl Evol.

[72] Giribet G, Edgecombe GD (2017). Current understanding of Ecdysozoa and its internal phylogenetic relationships. Integr Comp Biol.

[73] Dunn CW, Hejnol A, Matus DQ, Pang K, Browne WE, Smith SA, Seaver E, Rouse GW, Obst M, Edgecombe GD, Sørensen MV (2008). Broad phylogenomic sampling improves resolution of the animal tree of life. Nature..

[74] Hejnol A, Obst M, Stamatakis A, Ott M, Rouse GW, Edgecombe GD, Martinez P, Baguñà J, Bailly X, Jondelius U, Wiens M (2009). Assessing the root of bilaterian animals with scalable phylogenomic methods. Proc R Soc London B: Biol Sci.

[75] Borner J, Rehm P, Schill RO, Ebersberger I, Burmester T (2014). A transcriptome approach to ecdysozoan phylogeny. Mol Phylogenet Evol.

[76] Laumer CE, Bekkouche N, Kerbl A, Goetz F, Neves RC, Sørensen MV, Kristensen RM, Hejnol A, Dunn CW, Giribet G, Worsaae K (2015). Spiralian phylogeny informs the evolution of microscopic lineages. Curr Biol.

[77] Yoshida Y, Koutsovoulos G, Laetsch DR, Stevens L, Kumar S, Horikawa DD, Ishino K, Komine S, Kunieda T, Tomita M, Blaxter M (2017). Comparative genomics of the tardigrades *Hypsibius dujardini* and *Ramazzottius varieornatus*. PLoS Biol.

[78] Baylis HA, de Beer GR (1938). Helminths and evolution. Evolution.

[79] Dougherty EC (1951). Evolution of zooparasitic groups in the phylum Nematoda, with special reference to host-distribution. J Parasitol.

[80] Osche G (1956). Die Präadaptation freilebender Nematoden an den Parasitismus. Zool Anz.

[81] Anderson RC (1984). The origins of zooparasitic nematodes. Can J Zool.

[82] Clark WC (1994). Origins of the parasitic habit in the Nematoda. Int J Parasitol.

[83] Blaxter M (2003). Nematoda: genes, genomes and the evolution of parasitism. Adv Parasitol.

[84] Sudhaus W (1974). Nematoden (inbesondere Rhabditiden) des Strandanwurfs und ihre Beziehungen zu Krebsen. Faun.-ökol. Mitt..

[85] Tchesunov AV (2009). Diversity and forms of evolution of the nematodes associated with marine benthic invertebrates. Uchenye Zapiski Kazanskogo gosudarstvennogo universiteta, Estestvennye nauki.

[86] Sudhaus W. Evolution of insect parasitism in rhabditid and diplogastrid nematodes. In: Makarov SE, Dmitrijevic RN, editors. Advances in arachnology and developmental biology. Belgrade: SASA; 2008. p. 143–61. https://pdfs.semanticscholar.org/38df/94d708117d8fa4ace6db9ebd4d80fd5f8602.pdf.

[87] Sudhaus W (2010). Preadaptive plateau in Rhabditida (Nematoda) allowed the repeated evolution of zooparasites, with an outlook on evolution of life cycles within Spiroascarida. Palaeodiversity..

[88] Zarowiecki M, Berriman M (2015). What helminth genomes have taught us about parasite evolution. Parasitol..

[89] Viney M (2018). The genomic basis of nematode parasitism. Brief Funct Genomics.

[90] Weinstein Sara B., Kuris Armand M. (2016). Independent origins of parasitism in Animalia. Biology Letters.

[91] Spiridonov SE, Subbotin SA (2016). Phylogeny and phylogeography of *Heterorhabditis* and *Steinernema*. Nematology Monographs & Perspectives.

[92] Schmidt-Rhaesa A. Handbook of zoology. Gastrotricha, Cycloneuralia and Gnathifera. Volume 2: Nematoda: Walter de Gruyter GmbH; 2014. https://www.degruyter.com/view/product/180464.

[93] Poinar G, Lewis SC, Hagen NT, Hyman B (2011). Systematic affinity of the sea urchin parasite, *Echonomermella matsi* Jones & Hagen (Enoplida, Echonomermellidae). Nematology..

[94] Platonova TA, Potin VV. On new genera *Harpagonchus* and *Harpagonchoides* (Nematoda, Chromadorida, Harpagonchidae fam. N.) living on the parapodia and gills of the antarctic polychaetes Aglaophamus Kinberg and Hemipodus Quatrefages. Issledovania fauny morjei. 1972;11:81–87.

[95] Hopper BE (1966). *Theristus polychaetophilus* n. sp. (Nematoda), an external parasite of the spionid polychaete Scolelepis (Scolelepis) squamata (Müller, 1806). Can J Zool.

[96] Poinar G, Duarte D, Santos MJ (2010). *Halomonhystera parasitica* n. sp. (Nematoda: Monhysteridae), a parasite of *Talorchestia brito* (Crustacea: Talitridae) in Portugal. Syst Parasitol.

[97] Ivanova ES, Spiridonov SE (2011). Two new species of creagrocercid nematodes parasitic in earthworms, with comments on the phylogenetic affiliations of the Creagrocercidae Baylis, 1943. Syst Parasitol.

[98] Hope WD, Tchesunov AV (1999). *Smithsoninema inaequale* n. g, and n. sp. (Nematoda, Leptolaimidae) inhabiting the test of a foraminiferan. Invert Biol.

[99] Holovachov O, Boström S (2014). Swedish Plectida (Nematoda). Part 6. *Neocamacolaimus parasiticus* gen. N., sp. n. Zootaxa..

[100] Higgins RP, Thiel H. Introduction to the study of meiofauna. Washington D.C.: Smithsonian Institution Press; 1988. http://www.vliz.be/en/imis?module=ref&refid=34444&printversion=1&dropIMIStitle=1.

[101] Lee RYN, Howe KL, Harris TW, Arnaboldi V, Cain S, Chan J (2017). WormBase 2017: molting into a new stage. Nucleic Acids Res.

[102] Martin John, Rosa Bruce A., Ozersky Philip, Hallsworth-Pepin Kymberlie, Zhang Xu, Bhonagiri-Palsikar Veena, Tyagi Rahul, Wang Qi, Choi Young-Jun, Gao Xin, McNulty Samantha N., Brindley Paul J., Mitreva Makedonka (2014). Helminth.net: expansions to Nematode.net and an introduction to Trematode.net. Nucleic Acids Research.

[103] Chen S, Huang T, Zhou Y, Han Y, Xu M, Gu J (2017). AfterQC: automatic filtering, trimming, error removing and quality control for fastq data. BMC Bioinformatics.

[104] Grabherr MG, Haas BJ, Yassour M, Levin JZ, Thompson DA, Amit I (2011). Full-length transcriptome assembly from RNA-seq data without a reference genome. Nat Biotechnol.

[105] Afgan E, Baker D, van den Beek M, Blankenberg D, Bouvier D, Čech M (2016). The galaxy platform for accessible, reproducible and collaborative biomedical analyses: 2016 update. Nucleic Acids Res.

[106] Haas BJ, Papanicolaou A, Yassour M (2013). De novo transcript sequence reconstruction from RNA-seq using the trinity platform for reference generation and analysis. Nat Protoc.

[107] Finn RD, Coggill P, Eberhardt RY, Eddy SR, Mistry J, Mitchell AL (2016). The Pfam protein families database: towards a more sustainable future. Nuc Acids Res.

[108] Katoh K, Standley DM (2013). MAFFT multiple sequence alignment software version 7: improvements in performance and usability. Mol Biol Evol.

[109] Price MN, Dehal PS, Arkin AP (2010). FastTree 2--approximately maximum-likelihood trees for large alignments. PLoS One.

[110] Kück P, Longo GC (2014). FASconCAT-G: extensive functions for multiple sequence alignment preparations concerning phylogenetic studies. Front Zool.

[111] Stamatakis A (2014). RAxML version 8: a tool for phylogenetic analysis and post-analysis of large phylogenies. Bioinformatics.

[112] Nguyen LT, Schmidt HA, von Haeseler A, Minh BQ (2014). IQ-TREE: a fast and effective stochastic algorithm for estimating maximum-likelihood phylogenies. Mol Biol Evol.

[113] Lanfear R, Frandsen PB, Wright AM, Senfeld T, Calcott B (2016). PartitionFinder 2: new methods for selecting partitioned models of evolution for molecular and morphological phylogenetic analyses. Mol Biol Evol.

[114] Holovachov O (2016). Metabarcoding of marine nematodes – evaluation of similarity scores used in alignment-based taxonomy assignment approach. BDJ..

[115] Holovachov O (2016). Metabarcoding of marine nematodes – evaluation of reference datasets used in tree-based taxonomy assignment approach. BDJ..

[116] Quast C, Pruesse E, Yilmaz P, Gerken J, Schweer T, Yarza P, Peplies J, Glöckner FO (2013). The SILVA ribosomal RNA gene database project: improved data processing and web-based tools. Nucl Acids Res.

