# Supplementary figures and images for: Improved phylogenomic sampling of free-living nematodes enhances resolution of higher-level nematode phylogeny
Source: BMC Evol Biol. 2019 Jun 13;19:121. doi: 10.1186/s12862-019-1444-x (PMC6567515; doi:10.1186/s12862-019-1444-x)

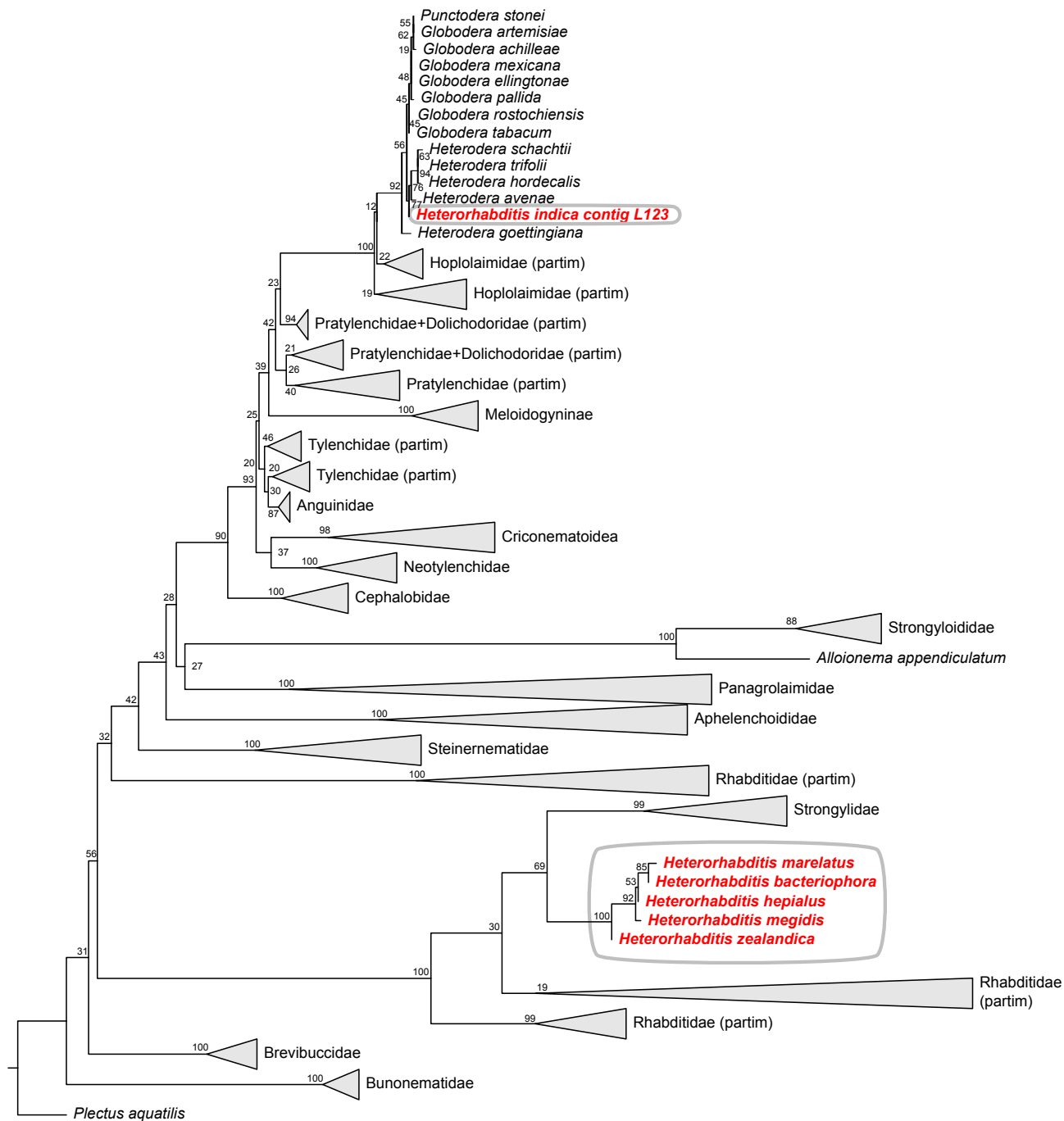

Supplement: Supplementary file 1 — Figure S1. Phylogenetic position of 18S rDNA fragment (contig Locus_123_Transcript_1/1) extracted from Heterorhabditis indica transcriptome dataset (http://insilico.iari.res.in/hindica/). (PDF 46 kb) [file 12862_2019_1444_MOESM1_ESM.pdf]
