# Supplementary material for: Improved phylogenomic sampling of free-living nematodes enhances resolution of higher-level nematode phylogeny
Source: BMC Evol Biol. 2019 Jun 13;19:121. doi: 10.1186/s12862-019-1444-x (PMC6567515; doi:10.1186/s12862-019-1444-x)
Supplement: Supplementary file 2 — Table S1. Names, classification (family and order), accession number (BioProject) or download link, and source/citation for publicly available genomes and transcriptomes of nematodes used in phylogenetic analysis. Classification is based on [1] with modifications [2, 3]. Table S2. Names and origin data or reference for publicly available genomes, transcriptomes or proteomes of non-nematode (outgroup) taxa. (DOCX 22 kb) [file 12862_2019_1444_MOESM2_ESM.docx]

**Supplementary Table 1**. Names, classification (family and order), accession number (BioProject) or download link, and source/citation for publicly available genomes and transcriptomes of nematodes used in phylogenetic analysis. Classification is based on [1] with modifications [2,3].

| Name | BioProject | | | Source | HaMStR orthologs |
| --- | --- | --- | --- | --- | --- |
| *Acanthocheilonema viteae*, Onchocercidae, Rhabditida | PRJEB4306 | | | wormbase.org, nematodes.org | 925 |
| *Ancylostoma caninum*, Ancylostomatidae, Rhabditida | PRJNA72585 | | | wormbase.org, nematode.net | 987 |
| *Ancylostoma ceylanicum*, Ancylostomatidae, Rhabditida | PRJNA231479 | | | [4], wormbase.org | 1013 |
| *Ancylostoma duodenale*, Ancylostomatidae, Rhabditida | PRJNA72581 | | | wormbase.org, nematode.net | 991 |
| *Angiostrongylus cantonensis*, Metastrongylidae, Rhabditida | PRJEB493 | | | wormbase.org | 990 |
| *Angiostrongylus costaricensis*, Metastrongylidae, Rhabditida | PRJEB494 | | | wormbase.org | 991 |
| *Anisakis simplex*, Anisakidae, Rhabditida | PRJEB496 | | | wormbase.org | 967 |
| *Aphelenchoides fragariae*, Aphelenchoididae, Rhabditida | PRJNA233627 | | | [5] | 996 |
| *Aphelenchoides ritzemabosi*, Aphelenchoididae, Rhabditida | PRJNA278017 | | | [6] | 960 |
| *Ascaris lumbricoides*, Ascarididae, Rhabditida | PRJEB4950 | | | wormbase.org | 996 |
| *Ascaris suum*, Ascarididae, Rhabditida | PRJNA62057 | | | [7], wormbase.org | 989 |
| *Brugia malayi*, Onchocercidae, Rhabditida | PRJNA10729 | | | [8], wormbase.org | 986 |
| *Brugia pahangi*, Onchocercidae, Rhabditida | PRJEB497 | | | wormbase.org | 953 |
| *Brugia timori*, Onchocercidae, Rhabditida | PRJEB4663 | | | wormbase.org | 947 |
| *Bursaphelenchus xylophilus*, Aphelenchoididae, Rhabditida | PRJEA64437 | | | [9], wormbase.org | 991 |
| *Caenorhabditis angaria*, Rhabditidae, Rhabditida | PRJNA51225 | | | [10], wormbase.org | 951 |
| *Caenorhabditis brenneri*, Rhabditidae, Rhabditida | PRJNA20035 | | | wormbase.org | 1010 |
| *Caenorhabditis briggsae*, Rhabditidae, Rhabditida | PRJNA10731 | | | [11], wormbase.org | 1011 |
| *Caenorhabditis elegans*, Rhabditidae, Rhabditida | PRJNA13758 | | | [12] | 1031 |
| *Caenorhabditis japonica*, Rhabditidae, Rhabditida | PRJNA12591 | | | wormbase.org | 1000 |
| *Caenorhabditis monodelphis*, Rhabditidae, Rhabditida |  | | | [13], download.caenorhabditis.org | 976 |
| *Caenorhabditis remanei*, Rhabditidae, Rhabditida | PRJNA53967 | | | [14], wormbase.org | 1020 |
| *Caenorhabditis sinica*, Rhabditidae, Rhabditida | PRJNA194557 | | | wormbase.org | 1013 |
| *Caenorhabditis tropicalis*, Rhabditidae, Rhabditida | PRJNA53597 | | | wormbase.org | 1003 |
| *Cylicostephanus goldi*, Strongylidae, Rhabditida | PRJEB498 | | | wormbase.org | 754 |
| *Deladenus siricidicola*, Neotylenchidae, Rhabditida | PRJNA46301 | | | www.ncbi.nlm.nih.gov | 829 |
| *Dictyocaulus viviparus*, Trichostrongylidae, Rhabditida | PRJEB5116 | | | wormbase.org, nematodes.org | 959 |
| *Diploscapter coronatus*, Rhabditidae, Rhabditida | PRJDB3143 | | | [15], wormbase org | 892 |
| *Diploscapter pachys*, Rhabditidae, Rhabditida | PRJNA280107 | | | [16], wormbase org | 968 |
| *Dirofilaria immitis*, Onchocercidae, Rhabditida | PRJEB1797 | | | [17], wormbase.org | 965 |
| *Ditylenchus destructor*, Anguinidae, Rhabditida | PRJNA312427 | | | [18], wormbase.org | 869 |
| *Dracunculus medinensis*, Dracunculidae, Rhabditida | PRJEB500 | | | wormbase.org | 969 |
| *Elaeophora elaphi*, Onchocercidae, Rhabditida | PRJEB502 | | | wormbase.org | 938 |
| *Enoplus brevis*, Enoplidae, Enoplida | PRJEB7588 | | | [19], www.ncbi.nlm.nih.gov | 1020 |
| *Enterobius vermicularis*, Oxyuridae, Rhabditida | PRJEB503 | | | wormbase.org | 988 |
| *Globodera pallida*, Hoplolaimidae, Rhabditida | PRJEB123 | | | [20], wormbase.org | 808 |
| *Globodera ellingtonae*, Hoplolaimidae, Rhabditida | PRJNA308318 | | | [21] | 886 |
| *Globodera rostochiensis*, Hoplolaimidae, Rhabditida | PRJEB13504 | | | [22], wormbase.org | 978 |
| *Gongylonema pulchrum*, Gongylonematidae, Rhabditida | PRJEB505 | | | wormbase.org | 965 |
| *Haemonchus contortus*, Trichostrongylidae, Rhabditida | PRJEB506 | | | [23], wormbase.org | 943 |
| *Haemonchus placei*, Trichostrongylidae, Rhabditida | PRJEB509 | | | wormbase.org | 992 |
| *Heligmosomoides polygyrus*, Trichostrongylidae, Rhabditida | PRJEB1203 | | | wormbase.org | 959 |
| *Heterorhabditis bacteriophora*, Heterorhabditidae, Rhabditida | PRJNA13977 | | | [24], wormbase.org | 824 |
| *Heterorhabditis indica*, Heterorhabditidae, Rhabditida | PRJEB10852 | | | [25] | 945 |
| *Litomosoides sigmodontis*, Onchocercidae, Rhabditida | PRJEB3075 | | | wormbase.org, nematodes.org | 957 |
| *Loa loa*, Onchocercidae, Rhabditida | PRJNA246086 | | | [26], nematodes.org | 950 |
| *Longidorus elongatus*, Longidoridae, Dorylaimida | PRJEB8328 | | | [27], www.ncbi.nlm.nih.gov | 998 |
| *Meloidogyne floridensis*, Hoplolaimidae, Rhabditida | PRJEB6016 | | | wormbase.org, nematodes.org | 947 |
| *Meloidogyne hapla*, Hoplolaimidae, Rhabditida | PRJNA29083 | | | [28], wormbase.org | 940 |
| *Meloidogyne incognita*, Hoplolaimidae, Rhabditida | PRJEA28837/PRJEB8714 | | | [29,30], wormbase.org | 795 |
| *Nacobbus aberrans*, Pratylenchidae, Rhabditida | PRJEB6762 | | | [31], nematode.net | 894 |
| *Necator americanus*, Ancylostomatidae, Rhabditida | PRJNA72135 | | | [32], wormbase.org | 973 |
| *Nippostrongylus brasiliensis*, Heligmonellidae, Rhabditida | PRJEB511 | | | wormbase.org | 985 |
| *Oesophagostomum dentatum*, Strongylidae, Rhabditida | PRJNA72579 | | | wormbase.org, nematode.net | 950 |
| *Onchocerca flexuosa*, Onchocercidae, Rhabditida | PRJEB512 | | | wormbase.org | 935 |
| *Onchocerca ochengi*, Onchocercidae, Rhabditida | PRJEB1204 | | | wormbase.org | 958 |
| *Onchocerca volvulus*, Onchocercidae, Rhabditida | PRJEB513 | | | [33], wormbase.org | 970 |
| *Oscheius tipulae*, Rhabditidae, Rhabditida | PRJEB15512 | | | [34], caenorhabditis.org | 973 |
| *Panagrellus redivivus*, Panagrolaimidae, Rhabditida | PRJNA186477 WS255 | | | [35], wormbase.org | 998 |
| *Panagrellus redivivus*, Panagrolaimidae, Rhabditida | PRJNA186477 WBSP6 | | | [35], wormbase.org | 998 |
| *Panagrolaimus davidi*, Panagrolaimidae, Rhabditida | SAMN02799240 | | | [36] | 862 |
| *Panagrolaimus superbus*, Panagrolaimidae, Rhabditida | SAMN00167950 | | | [37] | 224 |
| *Parascaris equorum*, Ascarididae, Rhabditida | PRJEB514 | | | wormbase.org | 773 |
| *Parastrongyloides trichosuri*, Strongyloididae, Rhabditida | PRJEB515 | | | [38], wormbase.org | 972 |
| *Plectus sambesii*, Plectidae, Plectida | PRJNA506962 | | | Schiffer & Kraus, unpublished | 1015 |
| *Prionchulus punctatus*, Mononchidae, Mononchida | PRJEB7585 | | | [19], www.ncbi.nlm.nih.gov | 1012 |
| *Pristionchus exspectatus*, Diplogastridae, Rhabditida | PRJEB6009 | | | [39], wormbase.org | 786 |
| *Pristionchus pacificus*, Diplogastridae, Rhabditida | PRJNA12644 | | | [40, 41], wormbase.org | 873 |
| *Pontonema vulgare*, Oncholaimidae, Enoplida | PRJNA504396 | | | Hejnol et al., unpublished | 1004 |
| *Rhabditophanes* sp. KR3021, Alloionematidae, Rhabditida | PRJEB1297 | | | [38], wormbase.org | 968 |
| *Romanomermis culicivorax*, Mermithidae, Mermithida | PRJEB1358 | | | [42], nematodes.org | 972 |
| *Soboliphyme baturini*, Soboliphymatidae, Dioctophymatida | PRJEB516 | | | wormbase.org | 929 |
| *Steinernema carpocapsae*, Steinernematidae, Rhabditida | PRJNA202318 | | | [43], wormbase.org | 1014 |
| *Steinernema feltiae*, Steinernematidae, Rhabditida | PRJNA204661 | | | [43], wormbase.org | 1005 |
| *Steinernema glaseri*, Steinernematidae, Rhabditida | PRJNA204943 | | | [43], wormbase.org | 1003 |
| *Steinernema monticolum*, Steinernematidae, Rhabditida | PRJNA205067 | | | [43], wormbase.org | 1002 |
| *Steinernema scapterisci*, Steinernematidae, Rhabditida | PRJNA204942 | | | [43], wormbase.org | 1006 |
| *Strongyloides papillosus*, Strongyloididae, Rhabditida | PRJEB525 | | | [38], wormbase.org | 991 |
| *Strongyloides ratti*, Strongyloididae, Rhabditida | PRJEB125 | | | [38], wormbase.org | 967 |
| *Strongyloides stercoralis*, Strongyloididae, Rhabditida | PRJEB528 | | | [38], wormbase.org | 975 |
| *Strongyloides venezuelensis*, Strongyloididae, Rhabditida | PRJEB530 | | | [38], wormbase.org | 960 |
| *Strongylus vulgaris*, Strongylidae, Rhabditida | PRJEB531 | | | wormbase.org | 873 |
| *Syphacia muris*, Oxyuridae, Rhabditida | PRJEB524 | | | wormbase.org | 970 |
| *Teladorsagia circumcincta*, Trichostrongylidae, Rhabditida | PRJNA72569 | | | wormbase.org, nematode.net | 989 |
| *Thelazia callipaeda*, Thelaziidae, Rhabditida | PRJEB1205 | | | wormbase.org | 960 |
| *Toxocara canis*, Ascarididae, Rhabditida | PRJEB533 | | | wormbase.org | 992 |
| *Trichinella britovi*, Trichinellidae, Trichinellida | PRJNA257433 | | | [44], wormbase.org | 839 |
| *Trichinella murrelli*, Trichinellidae, Trichinellida | PRJNA257433 | | | [44], wormbase.org | 842 |
| *Trichinella nativa*, Trichinellidae, Trichinellida | PRJNA179527 | | | wormbase.org, nematode.net | 858 |
| *Trichinella patagoniensis*, Trichinellidae, Trichinellida | PRJNA257433 | | | [44], wormbase.org | 848 |
| *Trichinella paupae*, Trichinellidae, Trichinellida | PRJNA257433 | | | [44], wormbase.org | 810 |
| *Trichinella pseudospiralis*, Trichinellidae, Trichinellida | PRJNA257433 | | | [44], wormbase.org | 820 |
| *Trichinella spiralis*, Trichinellidae, Trichinellida | PRJNA12603 | | | [45], wormbase.org, nematode.net | 853 |
| *Trichinella zimbabwensis*, Trichinellidae, Trichinellida | PRJNA257433 | | | [44], wormbase.org | 803 |
| *Trichuris muris*, Trichuridae, Trichinellida | PRJEB126 | | | [46], wormbase.org | 924 |
| *Trichuris suis*, Trichuridae, Trichinellida | PRJNA179528 | | | wormbase.org, nematode.net | 868 |
| *Trichuris trichiura*, Trichuridae, Trichinellida | PRJEB535 | | | [46], wormbase.org | 934 |
| *Wuchereria bancrofti*, Onchocercidae, Rhabditida | PRJEB536 | | | wormbase.org | 926 |
| *Xiphinema index*, Longidoridae, Dorylaimida | PRJEB22758 | | | [27], www.ncbi.nlm.nih.gov | 990 |

**Supplementary Table 2**. Names and origin data or reference for publicly available genomes, transcriptomes or proteomes of non-nematode (outgroup) taxa.

| **Name** | **BioProject/Source** | **References** | **HaMStR orthologs** |
| --- | --- | --- | --- |
| **Annelida** |  |  |  |
| *Capitella teleta* | JGI filtered gene models v1.1 | [47,48] | 1005 |
| **Arthropoda** |  |  |  |
| *Daphnia pulex* | JGI filtered gene models v1.1 | [47,48] | 994 |
| *Drosophila melanogaster* | HaMStR Model Organisms core ortholog set | [49] | 1021 |
| *Ephemera danica* | ftp.hgsc.bcm.edu | [50] | 1009 |
| *Eurytemora affinis* | ftp.hgsc.bcm.edu | [50] | 924 |
| *Hyalella azteca* | ftp.hgsc.bcm.edu | [50] | 910 |
| *Ixodes scapularis* | ftp.ensemblgenomes.org | [51] | 965 |
| *Lactrodectus hesperus* | ftp.hgsc.bcm.edu | [50] | 759 |
| *Limulus polyphemus* | NCBI SRR1145732 | [52] | 947 |
| *Loxosceles reclusa* | ftp.hgsc.bcm.edu | [50] | 714 |
| *Strigamia maritima* | ftp.ensemblgenomes.org | [53] | 940 |
| **Brachiopoda** |  |  |  |
| *Lingula anatina* | http://marinegenomics.oist.jp/lingula/download/lingula_transcriptome_v1.0_prot.fa.gz | [54] | 1011 |
| **Kinorhyncha** |  |  |  |
| *Pycnophyes kielensis* | SRR1141803 | [55] | 197 |
| **Mollusca** |  |  |  |
| *Crassostrea gigas* | gigadb.org/Pacific_oyster | [56] | 994 |
| *Lottia gigantea* | JGI filtered models v. 1.0 | [48] | 1005 |
| **Onychophora** |  |  |  |
| *Peripatopsis capensis* | NCBI SRR1145776 | [52] | 765 |
| **Priapulida** |  |  |  |
| *Halicryptus spinulosus* | NCBI SRR2682062 | [57] | 989 |
| *Priapulus caudatus* | NCBI SRR1800229 | [58] | 767 |
| *Priapulus* sp. | NCBI SRR1611567 | [59] | 772 |
| **Tardigrada** |  |  |  |
| *Hypsibius dujardini* | NCBI SRS1924217 | [60] | 975 |
| *Milnesium tardigradum* | NCBI SRR1046361, SRR1046360, SRR1046358, & SRR1046127 | [61] | 909 |
| *Ramazzottius varieornatus* | kumamushi.org/database.html | [62] | 986 |

**References**

1. De Ley P, Blaxter ML. A new system for Nematoda: combining morphological characters with molecular trees, and translating clades into ranks and taxa. Nematology Monographs and Perspectives. 2004;2:633-653.

2. Sudhaus W, Fürst von Lieven A. A phylogenetic classification and catalogue of the Diplogastridae (Secernentea, Nematoda). J Nematode Morphol Syst. 2003;6(1):43-90.

3. Sudhaus W. Phylogenetic systematisation and catalogue of paraphyletic “Rhabditidae” (Secernentea, Nematoda). J Nematode Morphol Syst. 2011;14(2):113-178.

4. Schwarz EM, Hu Y, Antoshechkin I, Miller MM, Sternberg PW, Aroian RV. The genome and transcriptome of the zoonotic hookworm *Ancylostoma ceylanicum* identify infection-specific gene families, Nat Genet. 2015;47(4):416-22.

5. Fu Z, Wells CE, Collier G, Agudelo P. *De novo* transcriptome assembly of the foliar nematode *Aphelenchoides fragariae*. J Nematol. 2012;44(4):462.

6. Xiang Y, Wang DW, Li J-Y, Xie H, Xu C-L, Li Y. Transcriptome analysis of the Chrysanthemum Foliar Nematode, *Aphelenchoides ritzemabosi* (Aphelenchida: Aphelenchoididae), PLoS One. 2016;11(11):e0166877.

7. Wang J, Gao S, Mostovoy Y, Kang Y, Zagoskin M, Sun Y, et al. Comparative genome analysis of programmed DNA elimination in nematodes. Genome Res. 2017;27(12):2001-2014.

8. Ghedin E, Wang S, Spiro D, Caler E, Zhao Q, Crabtree J, et al. Draft genome of the filarial nematode parasite *Brugia malayi*. Science. 2007;317(5845):1756-1760.

9. Kikuchi T, Cotton JA, Dalzell JJ, Hasegawa K, Kanzaki N, McVeigh P, et al. Genomic insights into the origin of parasitism in the emerging plant pathogen *Bursaphelenchus xylophilus*. PLoS Pathog. 2011;7(9):e1002219.

10. Mortazavi A, Schwarz EM, Williams B, Schaeffer L, Antoshechkin I, Wold BJ, Sternberg PW. Scaffolding a *Caenorhabditis* nematode genome with RNA-seq. Genome Res. 2010;20(12):1740-1747.

11. Stein LD, Bao Z, Blasiar D, Blumenthal T, Brent MR, Chen N, et al. The genome sequence of *Caenorhabditis briggsae*: a platform for comparative genomics. PLoS Biol. 2003;1(2):E45.

12. *C. elegans* Sequencing Consortium. Genome sequence of the nematode *C. elegans*: a platform for investigating biology. Science. 1998;282(5396):2012-2018.

13. Slos D, Sudhaus W, Stevens L, Bert W, Blaxter M. *Caenorhabditis monodelphis* sp. n.: defining the stem morphology and genomics of the genus *Caenorhabditis*. BMC Zoology. 2017;2:4

14. Jovelin R, Ajie BC, Phillips PC. Molecular evolution and quantitative variation for chemosensory behaviour in the nematode genus *Caenorhabditis*. Mol Ecol. 2003;12(5):1325-1337.

15. Hiraki H, Kagoshima H, Kraus C, Schiffer PH, Ueta Y, Kroiher M, et al. Genome analysis of *Diploscapter coronatus*: insights into molecular peculiarities of a nematode with parthenogenetic reproduction. BMC Genomics. 2017;18(1):478.

16. Fradin H, Kiontke K, Zegar C, Gutwein M, Lucas J, Kovtun M, et al. Genome architecture and evolution of a unichromosomal asexual nematode. Curr Biol. 2017;27(19):2928-2939.

17. Godel C, Kumar S, Koutsovoulos G, Ludin P, Nilsson D, Comandatore F, et al. The genome of the heartworm, *Dirofilaria immitis*, reveals drug and vaccine targets. FASEB J 2012;26(11):4650-4661.

18. Zheng J, Peng D, Chen L, Liu H, Chen F, Xu M, et al. The *Ditylenchus destructor* genome provides new insights into the evolution of plant parasitic nematodes. Proc Biol Sci. 2016;283:20160942.

19. Koutsovoulos G. Reconstructing the phylogenetic relationships of nematodes using draft genomes and transcriptomes. PhD [Dissertation]. Edinburgh, Scotland: University of Edinburgh; 2015.

20. Cotton JA, Lilley CJ, Jones LM, Kikuchi T, Reid AJ, Thorpe P, et al. The genome and life-stage specific transcriptomes of *Globodera pallida* elucidate key aspects of plant parasitism by a cyst nematode. Genome Biol. 2014;15(3):R43.

21. Phillips WS, Howe DK, Brown AMV, Eves-van den Akker S, Dettwyler L, Peetz AB, et al. The draft genome of *Globodera ellingtonae*. J Nematol. 2017;49(2):127-128.

22. Eves-van den Akker S, Laetsch DR, Thorpe P, Lilley CJ, Danchin EG, Da Rocha M, et al. The genome of the yellow potato cyst nematode, *Globodera rostochiensis*, reveals insights into the basis of parasitism and virulence. Genome Biol. 2016;17(1):124.

23. Laing R, Kikuchi T, Martinelli A, Tsai IJ, Beech RN, Redman E, et al. The genome and transcriptome of *Haemonchus contortus*, a key model parasite for drug and vaccine discovery. Genome Biol. 2013;14(8):R88.

24. Bai X, Adams BJ, Ciche TA, Clifton S, Gaugler R, Kim KS, et al. A lover and a fighter: the genome sequence of an entomopathogenic nematode *Heterorhabditis bacteriophora*. PLoS One. 2013;8(7):e69618.

25. Somvanshi VS, Gahoi S, Banakar P, Thakur PK, Kumar M, et al. A transcriptomic insight into the infective juvenile stage of the insect parasitic nematode, *Heterorhabditis indica*. BMC Genomics. 2016;17:166.

26. Tallon LJ, Liu X, Bennuru S, Chibucos MC, Godinez A, Ott S, et al. Single molecule sequencing and genome assembly of a clinical specimen of *Loa loa*, the causative agent of loiasis. BMC Genomics. 2014;15:788.

27. Danchin EGJ, Perfus-Barbeoch L, Rancurel C, Thorpe P, Da Rocha M, Bajew S, et al. The transcriptomes of *Xiphinema index* and *Longidorus elongatus* suggest independent acquisition of some plant parasitism genes by horizontal gene transfer in early-branching nematodes. Genes. 2017;8(10):287.

28. Opperman CH, Bird DM, Williamson VM, Rokhsar DS, Burke M, Cohn J, et al. Sequence and genetic map of *Meloidogyne hapla*: a compact nematode genome for plant parasitism. Proc Natl Acad Sci USA. 2008;105(39):14802-14807.

29. Abad P, Gouzy J, Aury JM, Castagnone-Sereno P, Danchin EG, Deleury E, et al. Genome sequence of the metazoan plant-parasitic nematode *Meloidogyne incognita*. Nat Biotechnol. 2008;26(8):909-915.

30. Blanc-Mathieu R, Perfus-Barbeoch L, Aury JM, Da Rocha M, Gouzy J, Sallet E, et al. Hybridization and polyploidy enable genomic plasticity without sex in the most devastating plant-parasitic nematodes. PLoS Genet. 2017;13(6):e1006777.

31. Eves-van den Akker S, Lilley CJ, Danchin EG, Rancurel C, Cock PJ, Urwin PE, Jones JT. The transcriptome of *Nacobbus aberrans* reveals insights into the evolution of sedentary endoparasitism in plant-parasitic nematodes. Genome Biol Evol. 2014;6(9):2181-94.

32. Tang YT, Gao X, Rosa BA, Abubucker S, Hallsworth-Pepin K, Martin J, et al. Genome of the human hookworm *Necator americanus*. Nat Genet. 2014;46(3):261-269.

33. Cotton JA, Bennuru S, Grote A, Harsha B, Tracey A, Beech R, et al. The genome of *Onchocerca volvulus*, agent of river blindness. Nat Microbiol. 2016;2:16216.

34. Besnard F, Koutsovoulos G, Dieudonné S, Blaxter M, Félix M-A. Toward universal forward genetics: using a draft genome sequence of the nematode *Oscheius tipulae* to identify mutations affecting vulva development. Genetics. 2017;206(4):1747-1761.

35. Srinivasan J, Dillman AR, Macchietto MG, Heikkinen L, Lakso M, Fracchia KM, et al. The draft genome and transcriptome of *Panagrellus redivivus* are shaped by the harsh demands of a free-living lifestyle. Genetics. 2013;193(4):1279-95.

36. Thorne MA, Kagoshima H, Clark MS, Marshall CJ, Wharton DA. Molecular analysis of the cold tolerant Antarctic nematode, *Panagrolaimus davidi*. PLoS One. 2014;9(8):e104526.

37. Tyson T, O'Mahony Zamora G, Wong S, Skelton M, Daly B, Jones JT, et al. A molecular analysis of desiccation tolerance mechanisms in the anhydrobiotic nematode *Panagrolaimus superbus* using expressed sequenced tags. BMC Res Notes. 2012;5:68.

38. Hunt VL, Tsai IJ, Coghlan A, Reid AJ, Holroyd N, Foth BJ, et al. The genomic basis of parasitism in the *Strongyloides* clade of nematodes. Nat Genet. 2016;48(3):299-307.

39. Rödelsperger C, Neher RA, Weller AM, Eberhardt G, Witte H, Mayer WE, et al. Characterization of genetic diversity in the nematode *Pristionchus pacificus* from population-scale resequencing data. Genetics. 2014;196(4):1153-1165.

40. Dieterich C, Clifton SW, Schuster LN, Chinwalla A, Delehaunty K, Dinkelacker I, et al. The *Pristionchus pacificus* genome provides a unique perspective on nematode lifestyle and parasitism. Nat Genet. 2008;40(10):1193-1198.

41. Rödelsperger C, Meyer JM, Prabh N, Lanz C, Bemm F, Sommer RJ. Single-molecule sequencing reveals the chromosome-scale genomic architecture of the nematode model organism *Pristionchus pacificus*. Cell Rep. 2017;21(3):834-844.

42. Schiffer PH, Kroiher M, Kraus C, Koutsovoulos GD, Kumar S, Camps JI, et al. The genome of *Romanomermis culicivorax*: revealing fundamental changes in the core developmental genetic toolkit in Nematoda. BMC Genomics. 2013;14:923.

43. Dillman AR, Macchietto M, Porter CF, Rogers A, Williams B, Antoshechkin I, et al. Comparative genomics of *Steinernema* reveals deeply conserved gene regulatory networks. Genome Biol. 2015;16:200.

44. Korhonen PK, Pozio E, La Rosa G, Chang BC, Koehler AV, Hoberg EP, et al. Phylogenomic and biogeographic reconstruction of the *Trichinella* complex. Nat Commun. 20161;7:10513.

45. Mitreva M, Jasmer DP, Zarlenga DS, Wang Z, Abubucker S, Martin J, et al. The draft genome of the parasitic nematode *Trichinella spiralis*. Nat Genet. 2011;43(3):228-235.

46. Foth BJ, Tsai IJ, Reid AJ, Bancroft AJ, Nichol S, Tracey A, et al. Whipworm genome and dual-species transcriptome analyses provide molecular insights into an intimate host-parasite interaction. Nat Genet. 2014;46(7):693-700.

47. Grigoriev IV, Nordberg H, Shabalov I, Aerts A, Cantor M, Goodstein D, et al. The genome portal of the Department of Energy Joint Genome Institute. Nucleic Acids Res. 2012;40:D26-32.

48. Nordberg H, Cantor M, Dusheyko S, Hua S, Poliakov A, Shabalov I, et al. The genome portal of the Department of Energy Joint Genome Institute: 2014 updates. Nucleic Acids Res. 2014;42(1):D26-31.

49. Sonnhammer ELL, Östlund G. InParanoid 8: orthology analysis between 273 proteomes, mostly eukaryotic. Nucleic Acids Res. 2015;43:D234-D239.

50. i5K Consortium. The i5K Initiative: Advancing arthropod genomics for knowledge, human health, agriculture, and the environment. J Hered. 2013;104(5):595–600.

51. Van Zee J, Geraci N, Guerrero F, Wikel S, Stuart J, Nene V et al. Tick genomics: the *Ixodes* genome project and beyond. Int J Parasitol 2007;37(12):1297-1305.

52. Sharma PP, Kaluziak ST, Pérez-Porro AR, González VL, Hormiga G, Wheeler WC, et al. Phylogenomic interrogation of Arachnida reveals systemic conflicts in phylogenetic signal. Mol Biol Evol. 2014;31(11):2963–2984.

53. Chipman AD, Ferrier DEK, Brena C, Qu J, Hughes DST, Schröder R et al. The first myriapod genome sequence reveals conservative arthropod gene content and genome organisation in the centipede *Strigamia maritima*. PLoS Biology 2014;12(11):e1002005.

54. Luo YJ, Takeuchi T, Koyanagi R, Yamada L, Kanda M, Khalturina M, Fujie M, Yamasaki S, Endo K, Satoh N. The *Lingula* genome provides insights into brachiopod evolution and the origin of phosphate biomineralization. Nat Comm. 2015;6:8301.

55. Borner J, Rehm P, Schill RO, Ebersberger I, Burmester T. A transcriptome approach to ecdysozoan phylogeny. Mol. Phylogenet. Evol. 2014;80:79-87.

56. Zhang G, Fang X, Guo X, Li L, Luo R, Xu F, et al. The oyster genome reveals stress adaptation and complexity of shell formation. Nature 2012; 490:49-54.

57. Cannon JT, Vellutini BC, Smith J, Ronquist F, Jondelius U, Hejnol A. Xenacoelomorpha is the sister group to Nephrozoa. Nature 2016; 530:89-93.

58. Egger B, Lapraz F, Tomiczek B, Müller S, Dessimoz C, Girstmair J, Škunca N, Rawlinson KA, Cameron CB, Beli E, Todaro MA, Gammoudi M, Noreña C, Telford MJ. A transcriptomic-phylogenomic analysis of the evolutionary relationships of flatworms. Curr Biol. 2015;25(10):1347-1353.

59. Halanych KM, Kocot KM. Repurposed transcriptomic data facilitate discovery of innate immunity toll-like receptor (TLR) genes across lophotrochozoa. Biol Bull. 2014;227:201-2019.

60. Yoshida Y, Koutsovoulos G, Laetsch DR, Stevens L, Kumar S, Horikawa DD, Ishino K, Komine S, Kunieda T, Tomita M, Blaxter M. Comparative genomics of the tardigrades *Hypsibius* *dujardini* and *Ramazzottius varieornatus*. PLoS biology. 2017;15(7):e2002266.

61. Wang C, Grohme MA, Mali B, Schill RO, Frohme M. Towards decrypting cryptobiosis--analyzing anhydrobiosis in the tardigrade *Milnesium tardigradum* using transcriptome sequencing. *PLoS One*. 2014;9(3):e92663.

62. Hashimoto T, Horikawa DD, Saito Y, Kuwahara H, Kozuka-Hata H, Shin-I T, et al. Extremotolerant tardigrade genome and improved radiotolerance of human cultured cells by tardigrade-unique protein. Nat Comm. 2016;7:12808.
